# High-Throughput Mutation Profiling Changes before and 3 Weeks after Chemotherapy in Newly Diagnosed Breast Cancer Patients

**DOI:** 10.1371/journal.pone.0142466

**Published:** 2015-12-02

**Authors:** Sing-Huang Tan, Nur Sabrina Sapari, Hui Miao, Mikael Hartman, Marie Loh, Wee-Joo Chng, Philip Iau, Shaik Ahmad Buhari, Richie Soong, Soo-Chin Lee

**Affiliations:** 1 Department of Haematology-Oncology, National University Cancer Institute Singapore, National University Health System, Singapore, Singapore; 2 Cancer Science Institute of Singapore, National University of Singapore, Singapore, Singapore; 3 Saw Swee Hock School of Public Health, National University of Singapore, Singapore, Singapore; 4 Department of Surgery, National University Cancer Institute Singapore, National University Health System, Singapore, Singapore; 5 Department of Pathology, National University of Singapore, Singapore, Singapore; Yale University, UNITED STATES

## Abstract

**Background:**

Changes in tumor DNA mutation status during chemotherapy can provide insights into tumor biology and drug resistance. The purpose of this study is to analyse the presence or absence of mutations in cancer-related genes using baseline breast tumor samples and those obtained after exposure to one cycle of chemotherapy to determine if any differences exist, and to correlate these differences with clinical and pathological features.

**Methods:**

Paired breast tumor core biopsies obtained pre- and post-first cycle doxorubicin (*n* = 18) or docetaxel (*n* = 22) in treatment-naïve breast cancer patients were analysed for 238 mutations in 19 cancer-related genes by the Sequenom Oncocarta assay.

**Results:**

Median age of patients was 48 years (range 32–64); 55% had estrogen receptor-positive tumors, and 60% had tumor reduction ≥25% after cycle 1. Mutations were detected in 10/40 (25%) pre-treatment and 11/40 (28%) post-treatment samples. Four mutation pattern categories were identified based on tumor mutation status pre- → post-treatment: wildtype (WT)→WT, *n* = 24; mutant (MT)→MT, *n* = 5; MT→WT, *n* = 5; WT→MT, *n* = 6. Overall, the majority of tumors were WT at baseline (30/40, 75%), of which 6/30 (20%) acquired new mutations after chemotherapy. Pre-treatment mutations were predominantly in *PIK3CA* (8/10, 80%), while post-treatment mutations were distributed in *PIK3CA*, *EGFR*, *PDGFRA*, *ABL1* and *MET*. All 6 WT→MT cases were treated with docetaxel. Higher mutant allele frequency in baseline MT tumors (n = 10; *PIK3CA* mutations n = 8) correlated with less tumor reduction after cycle 1 chemotherapy (R = -0.667, p = 0.035). No other associations were observed between mutation pattern category with treatment, clinicopathological features, and tumor response or survival.

**Conclusion:**

Tumor mutational profiles can change as quickly as after one cycle of chemotherapy in breast cancer. Understanding of these changes can provide insights on potential therapeutic options in residual resistant tumors.

**Trial Registration:**

ClinicalTrials.gov NCT00212082

## Introduction

Carcinogenesis is a multi-step process characterized by acquisition of molecular alterations in various signaling pathways involved in growth and development. Activation of oncogenes leading to cancer occurs via mechanisms such as gene amplification, chromosomal translocations, and point mutations that enhance the function of the "oncoprotein"[1]. Aberrations in oncogenes may be important in natural selection during tumorigenesis or upon treatment, and knowledge of clinically relevant mutations can aid tumor classification as well as facilitate patient stratification for deployment of targeted therapeutics.

Neoadjuvant chemotherapy for breast tumors provides a unique opportunity for acquisition of early information on *in vivo* tumor response and disease biology, and also lends an ideal model to evaluate biomarkers as the primary tumor may be sampled readily and repeatedly.

Chemotherapy-induced gene expression changes in breast tumors after one cycle or even as early as 24 hours after chemotherapy have been well described [2,3,4], but studies evaluating mutation shifts during neoadjuvant chemotherapy are at present very limited. A recent study using exome sequencing revealed that loss of *PIK3CA* and *TP53* mutations in breast tumors after 3–6 cycles of neoadjuvant chemotherapy translated to improved clinical responses and survival outcomes [5].

In order to gain an improved perspective into earlier molecular changes following chemotherapy, we sought to characterize the mutation profile of treatment-naïve primary breast tumors pre-treatment and 3 weeks after first exposure to doxorubicin or docetaxel chemotherapy using a high-throughput mutation analysis assay interrogating 238 mutations in 19 cancer-related genes [6]. We found distinct changes in mutation patterns pre- and post-treatment which allowed us to categorize our samples into 4 subgroups according to mutation profile variations with treatment. The mutation patterns were also assessed for their correlation with clinical parameters including chemotherapy type, estrogen receptor (ER) status, disease extent, tumor response, progression-free and overall survival.

## Materials and Methods

### Patients and tissues

Breast tumor samples were prospectively collected as part of a phase II randomized study that recruited Southeast Asian patients with newly diagnosed histologically or cytologically proven clinical stage II-IV breast cancer with measurable primary tumor in order to discover gene expression profiles that predicted for chemosensitivity (ClinicalTrials.gov ID: NCT00212082; S1 Text) [4]. This study was approved by the National Healthcare Group Domain Specific Review Board institutional ethics review committee, and all participants provided written informed consent. Patients were recruited from our institution and randomised at a ratio of 1:1 to one of two alternating sequences of doxorubicin (A) and docetaxel (T), starting with either doxorubicin 75mg/m^2^ or docetaxel 75mg/m^2^ every 3 weeks for 6 cycles (Arm A: A→T→A→T→A→T; Arm B: T→A→T→A→T→A) as primary systemic therapy. One to two core biopsies from the primary breast tumor were taken each at baseline and approximately 3 weeks after the first cycle of chemotherapy, snap frozen in liquid nitrogen and stored at -80°C until analysis. Only RNA was extracted from the first tumor core for gene expression analysis, the data of which has been reported previously [4,7], while both DNA and RNA was extracted from the second tumor core if available, for DNA mutational analysis and other RNA expression work. Bidimensional breast tumor assessments were performed at every cycle. Tumors were deemed to be intrinsically sensitive or resistant to cycle 1 chemotherapy if they demonstrated ≥25% or < 25% reduction in tumor dimensions respectively after cycle 1. After 6 cycles, the overall response rates (ORR) were classified as complete response, partial response, stable disease, or progressive disease in accordance with World Health Organisation criteria [8]. The study was approved by the institutional ethics review board and all patients provided written informed consent.

### Mutational analysis of invasive tumor

Tumor DNA was extracted from frozen tissues using Allprep DNA/RNA/Protein mini kit (Qiagen, Hilden, Germany), according to the manufacturer’s protocol. The quality and quantity of DNA was assessed by spectrophotometer on the Nanodrop 2000c (Wilmington, DE) but no microdissection was done. DNA mutation analysis was performed using the pre-designed OncoCarta™ mutation panel (Sequenom, San Diego, CA; S1 Table) on the MassARRAY Analyzer Compact (Sequenom) according to the manufacturer’s protocols. Each analysis consisted of 251 assays involving 24 pools of primer pairs and extension primers, and interrogated 238 non-synonymous mutations in 19 cancer-related genes [6]. Five-hundred nanograms of DNA from each patient sample were used for the assay. Mutations calls for each sample were determined using MassArray Typer Analyzer software 4.0.4.20 (Sequenom), and were identified by comparing ratios of the spectral wild type peaks to that of all suspected mutants. The RKO cell line containing the mutations *BRAF V600E* and *PIK3CA H1047R* was used as a positive control and included in every run. Associations were evaluated in relation to clinicopathological features such as type of chemotherapy exposure, age, race, presence or absence of metastases, ER status, post-cycle 1 tumor response and overall treatment responses. HER2/neu testing was only carried out in 4 samples and hence was not used in the analysis. We also analysed mutant allele frequency in baseline mutant tumors in relation to tumor responses post-cycle 1 chemotherapy and overall response after 6 cycles of chemotherapy.

### Statistical Analysis

For analysis of association with clinicopathological features, cases were divided into two groups consisting of those with no mutations in pre- and post-chemotherapy samples (WT→WT), and a group consisting of all other cases, including those with mutations in both pre- and post-chemotherapy samples (MT→MT), those with mutations in pre-chemotherapy samples but no mutations in post-chemotherapy samples (MT→WT), and those with no mutations in pre-chemotherapy samples but with mutations in post-chemotherapy samples (WT→MT). Associations with categorical variables were examined using Fisher’s exact test. Associations with age as a continuous variable were analyzed using the two-sample t-test.

Logistic regression was carried out to determine the association between mutational transition (MT→WT or WT→MT) and type of chemotherapy exposure (treatment arm A vs arm B) while adjusting for pre-chemotherapy mutation profile, presence of distant metastases and ER status.

Pearson's chi-squared test was used to evaluate the association between the types of mutations (*PIK3CA* vs others) in the 10 baseline mutant samples with respect to tumour response post-cycle 1 and after 6 cycles of chemotherapy (overall response). Pearson's chi-squared test was also used to determine the relation between high versus low mutant allele frequency (≥25% vs <25%) in the 10 baseline mutant samples and tumor response post-cycle 1 and overall response. A Pearson correlation coefficient test was carried out between the absolute tumour reduction (or increase) after one cycle of chemotherapy and baseline sample mutation allele frequency in baseline mutant tumors.

Progression-free survival (PFS) and overall survival (OS) of the WT→WT subgroup was compared with the other subgroups (MT→MT, MT→WT or WT→MT) using the Kaplan Meier method and logrank test. Multivariate Cox regression analyses were performed to test the effect of mutation subgroup type (WT→WT versus the rest) on PFS and OS while adjusting for T stage (T3 versus T4), presence of metastases (Yes versus No), ER and progesterone receptor (PgR) status (positive versus negative). All statistical analyses were two-sided, and performed using IBM SPSS Statistics Desktop 20 software (IBM, Armonk, NY). A p-value less than 0.05 was considered statistically significant.

## Results

### Clinical Characteristics

**Fig 1:**
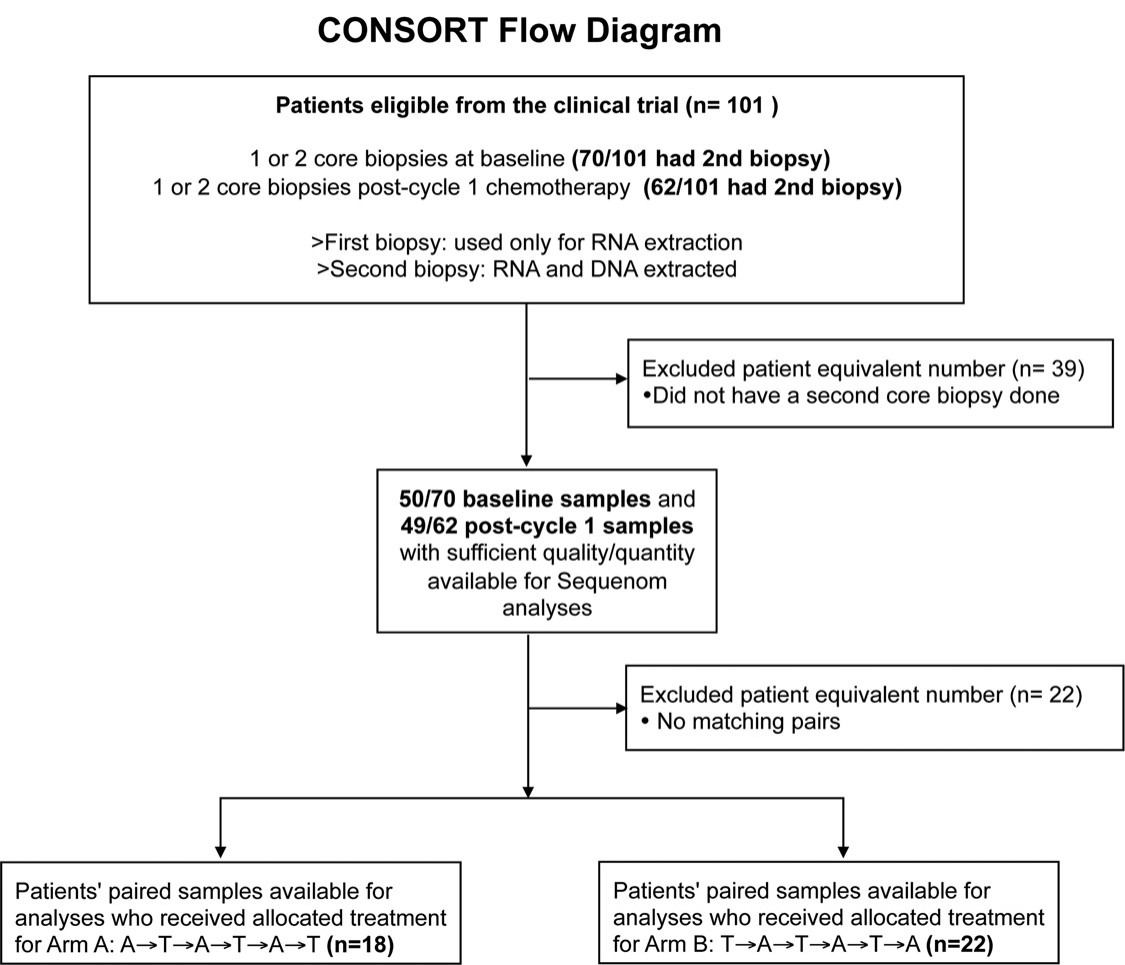
CONSORT flow chart in which the number of paired samples with data available for Sequenom analysis are shown.

Matched pre- and post-chemotherapy tumor DNA samples from 40 patients who were randomised between 26th April 2002 and 3^rd^ June 2005 were available for mutation analyses (Arm A: *n* = 18, Arm B: *n* = 22). Sixty-one patients’ paired samples were not feasible for analysis either due to unavailability of samples (n = 39) or inadequate DNA content (n = 22; Fig 1). An accompanying paraffin-embedded sample was taken at each time point, and the average cellularity of the baseline samples was 63% (range 10–95%), and that for the post-cycle 1 chemotherapy samples was 42% (range 10–90%).

None of the participants were lost to follow-up with the last follow-up date being 18^th^ February 2014.

The median age of the cohort was 48 years (range 32–64); 75% were Chinese (*n* = 30), 23% were Malay (*n* = 9) and one was Indian. Twenty-five percent (10/40) of the patients had metastatic disease at diagnosis, 28% (11/40) and 72% (29/40) had clinical T3 and T4 disease respectively, and 55% (22/40) had ER-positive tumors. Sixty percent of patients (24/40) achieved at least 25% tumor reduction after cycle 1 chemotherapy. After 6 cycles of chemotherapy, the overall objective clinical responses were as follows: complete clinical response (4/40; 10%); partial response (28/40; 70%); stable disease (7/40, 18%); one was non-evaluable. At the time of analysis, median follow-up was 74.5 months. Median PFS and OS of the entire cohort was 30 months and 75 months respectively.

### Mutation Analysis

**Fig 2:**
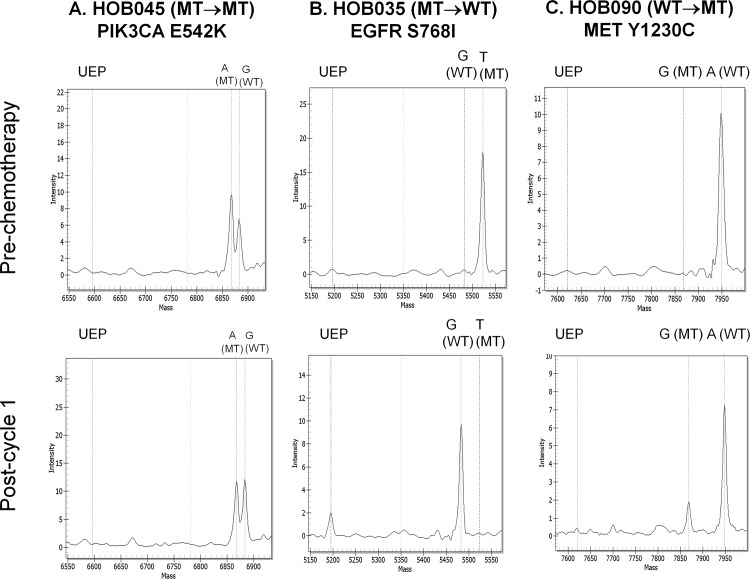
Oncogenic mutations detected pre- and post-chemotherapy. Representative chromatograms of (A) *PIK3CA E542K* in a MT→MT case HOB045, (B) *EGFR S768I* in a MT→WT case HOB035, and (C) *MET Y1230C* detected in a WT→MT case HOB090. The expected positions for the unextended primer (UEP) and the nucleotide and mutation status (mutant [MT] or wildtype [WT]) based on the size of the extension products are indicated above the gray vertical dashed lines.

Mutations were detected in 25% (10/40) of pre-treatment samples, with the majority being in *PIK3CA* (*n* = 8: *H1047R*, n = 3; *H1047L*, n = 2; *E542K*, n = 1; E545K, n = 1; *N345K*, n = 1), and the others being in *EGFR S768I* (n = 1) and *KIT Y503-F504insAY* (n = 1) (Fig 2, Table 1). A total of 28% (11/40) of post-treatment samples had mutations, the majority also being in *PIK3CA* (n = 6: *H1047R* n = 3; *E542K* n = 1; E545K n = 1; *N345K* n = 1), followed by *EGFR* (n = 2: *H773-V774insH* and *N771-P772>SVDNR*), *PDGFRA I843-S847>T* (n = 1), *ABL Y253H* (n = 1), and *MET Y1230C* (n = 1).

**Table 1:** Study cases according to mutation status in pre- and post-chemotherapy samples and their clinicopathological and tumor response characteristics (n = 40 pairs).

Category/Case	Mutation	Pre^a^/mutant allele frequency (if applicable)	Post^b^/mutant allele frequency (if applicable)	Arm^c^	Age^d^	Race	Baseline T stage	Met	ER	PgR	C1	ORR
**WT**→**WT cases**												
HOB002	none	WT	WT	A	47	Malay	T4	-	+	+	+	PR
HOB005	none	WT	WT	B	55	Chinese	T4	-	-	-	+	NE
HOB007	none	WT	WT	B	63	Chinese	T4	-	-	-	+	PR
HOB014	none	WT	WT	A	47	Chinese	T4	-	+	+	+	PR
HOB015	none	WT	WT	A	51	Chinese	T4	-	-	-	+	PR
HOB019	none	WT	WT	B	64	Malay	T4	+	+	-	-	SD
HOB027	none	WT	WT	B	45	Chinese	T3	-	-	-	-	SD
HOB028	none	WT	WT	B	40	Malay	T4	-	-	-	+	PR
HOB031	none	WT	WT	A	48	Chinese	T4	-	+	-	+	PR
HOB036	none	WT	WT	B	46	Chinese	T4	-	+	-	-	SD
HOB042	none	WT	WT	B	59	Indian	T4	+	-	-	-	SD
HOB043	none	WT	WT	A	44	Chinese	T4	-	-	-	+	PR
HOB046	none	WT	WT	B	44	Chinese	T4	+	+	-	+	PR
HOB054	none	WT	WT	A	43	Chinese	T3	-	+	+	+	CR
HOB055	none	WT	WT	B	43	Chinese	T4	-	+	+	+	PR
HOB060	none	WT	WT	A	36	Chinese	T3	-	-	-	+	PR
HOB064	none	WT	WT	A	38	Chinese	T3	-	-	-	-	SD
HOB069	none	WT	WT	A	37	Chinese	T3	-	+	-	-	PR
HOB071	none	WT	WT	A	56	Malay	T4	+	-	+	-	CR
HOB073	none	WT	WT	B	45	Malay	T4	-	+	+	+	PR
HOB084	none	WT	WT	A	45	Malay	T4	-	-	+	-	PR
HOB089	none	WT	WT	B	49	Chinese	T4	+	+	+	-	PR
HOB091	none	WT	WT	A	63	Chinese	T4	-	+	+	-	PR
HOB099	none	WT	WT	B	32	Chinese	T3	-	+	+	+	PR
**MT**→**MT cases**												
HOB062	*PIK3CA H1047R*	MT/18%	MT/24%	A	64	Chinese	T4	-	+	+	+	PR
HOB085	*PIK3CA H1047R*	MT/26%	MT/19%	A	64	Chinese	T4	-	+	+	-	PR
HOB045	*PIK3CA E542K*	MT/60%	MT/50%	B	40	Chinese	T3	-	+	+	+	PR
HOB056	*PIK3CA E545K*	MT/41%	MT/100%	A	44	Chinese	T4	+	+	+	+	CR
HOB063	*PIK3CA N345K*	MT/50%	MT/78%	A	56	Chinese	T4	-	-	+	-	PR
**MT**→**WT cases**												
HOB076	*PIK3CA H1047R*	MT/9%	WT	A	34	Chinese	T4	-	+	+	+	CR
HOB026	*PIK3CA H1047L*	MT/12%	WT	A	54	Chinese	T4	-	+	+	+	PR
HOB044	*PIK3CA H1047L*	MT/66%	WT	B	48	Malay	T3	-	-	-	-	PR
HOB035	*EGFR S768I*	MT/98%	WT	B	56	Malay	T4	+	-	-	-	SD
HOB086	*KIT Y503-F504insAY*	MT/20%	WT	B	51	Chinese	T3	-	-	-	+	PR
**WT**→**MT cases**												
HOB096	*ABL Y253H*	WT	MT/24%	B	56	Chinese	T3	-	-	-	-	PR
HOB088	*EGFR H773-V774insH*	WT	MT/51%	B	45	Chinese	T4	+	-	-	+	PR
HOB077	*EGFR N771-P772>SVDNR*	WT	MT/100%	B	63	Chinese	T4	+	+	-	-	SD
HOB090	*MET Y1230C*	WT	MT/20%	B	61	Chinese	T4	-	+	-	+	PR
HOB052	*PDGFRA I843-S847>T*	WT	MT/13%	B	54	Chinese	T4	+	-	-	+	PR
HOB080	*PIK3CA H1047R*	WT	MT/26%	B	58	Malay	T3	-	+	-	+	PR

**Abbreviations:** C1, + = tumor response ≥25% after cycle 1 chemotherapy, C1,— = tumour response <25% after cycle 1 chemotherapy; CR, complete response; ER, estrogen receptor status; Met, Presence of metastasis; MT, mutant; NE, non evaluable (patient had already started on another line of treatment); ORR, overall response rate; PgR, progesterone receptor; PR, partial response; SD, stable disease; T3, >50mm in greatest dimension; T4, tumor of any size with direct extension to the chest wall and/or to the skin (ulceration or skin nodules); WT, wildtype; -, negative/no; +, positive/yes

^a^ Status of respective mutation in pre-chemotherapy sample

^b^ Status of respective mutation in post-chemotherapy sample

^c^ Arm A, randomized to receive doxorubicin in first cycle; Arm B, randomized to receive docetaxel in first cycle

^d^ Age in years

Based on tumor mutation status before and after one cycle of chemotherapy, patient samples were divided into 4 categories: (1) WT→WT (24/40, 60%), (2) MT→MT (5/40, 13%), (3) MT→WT (5/40, 13%), (4) WT→MT (6/40, 15%). All samples with detectable mutations harboured only a single mutation. Of the 5 tumor pairs that had mutations before and after chemotherapy (MT→MT subgroup), the pre and post-chemotherapy mutations were identical in each tumor, and all mutations were in *PIK3CA (H1047R* n = 2; *E542K* n = 1; *E545K* n = 1; *N345K* n = 1*)*. There were 5 MT→WT cases, consisting of 3 cases that originally carried *PIK3CA* mutations (*H1047R* n = 1; *H1047L* n = 2), and 2 with an *EGFR S768I* and *KIT Y503-F504insAY* mutations respectively. A WT→MT pattern occurred in 6 cases; with the post-treatment mutations being *ABL1 Y253H*, *EGFR H773-V774insH*, *EGFR N771-P772>SVDNR*, *MET Y1230C*, *PDGFRA I843-S847>T*, and *PIK3CA H1047R*, respectively. The observed mutant allele frequencies are listed in Table 1. The mean mutant allele frequency in the 10 pre-treatment MT tumor samples was 40% (range 9–98%), while the mutant allele frequency in the 11 post-treatment MT tumor samples was 47.9% (range 13–100%).

### Association with Clinical Features

The distribution of exposure to either first-cycle doxorubicin or docetaxel were well balanced in the WT→WT (doxorubicin: 12 patients; docetaxel: 12 patients), and MT→WT groups (doxorubicin: 2 patients; docetaxel: 3 patients). However, 4 out of 5 patients were exposed to doxorubicin in the MT→MT subgroup, while all 6 patients in the WT→MT subgroup were treated initially with docetaxel. ER-status (positive versus negative) was distributed relatively evenly in all groups except in the MT→MT subgroup in which all but one tumor pair with *PIK3CA* mutations were ER-positive. The tumor sample harbouring a *PIK3CA* mutation that was ER-negative was progesterone receptor (PgR)-positive. In the MT→WT category, all the pre-treatment samples with *PIK3CA* mutations were ER-positive except one that was ER- and PgR-negative. Thus, 7 out of 9 paired samples with *PIK3CA* mutations in pre- and/or post-treatment samples were ER-positive.

No significant difference was observed between WT→WT and the combination of the other 3 subgroups (MT→MT, MT→WT, WT→MT) with respect to type of chemotherapy exposure, age, race, presence or absence of metastases, ER status, post-cycle 1 tumor responses and overall treatment responses (Table 2).

**Table 2:** Association of pre- and post-chemotherapy mutation patterns with clinicopathological features.

Feature	Wildtype (WT)→WT Cases	Other Cases^a^	*P* value^b^
Total	N = 24	N = 16	
**Treatment Arm**			
A	12 (50%)	6 (38%)	0.526
B	12 (50%)	10 (63%)	
**Age in Years**			
Mean±Standard Deviation	47.5±8.7	53.0±8.8	0.059^c^
**Race**			
Chinese	17 (71%)	13 (81%)	0.711
Non-Chinese	7 (29%)	3 (19%)	
**Metastases**			
Yes	5 (21%)	5 (31%)	0.482
No	19 (79%)	11(69%)	
**Estrogen Receptor Status**			
Positive	13 (54%)	9 (56%)	1.000
Negative	11 (46%)	7 (44%)	
**Cycle 1 Response ≥25%**			
Yes	14 (58%)	10 (63%)	0.528
No	10 (42%)	6 (38%)	
**Overall Response Rate** ^d^			
Complete Response (CR)	2 (8%)	2 (13%)	
Partial Response (PR)	16 (67%)	12 (75%)	0.678^e^
Stable Disease (SD)	5 (21%)	2 (13%)	

^a^ Mutant→Mutant, Mutant→Wildtype, Wildtype→Mutant cases

^b^ Fisher’s exact test, two-sided *P* value

^c^ Two-sample t-test, two-sided *P* value

^d^ One WT→WT was not evaluable for overall response

^e^ *P* value from analysis of CR/PR vs SD

**Fig 3:**
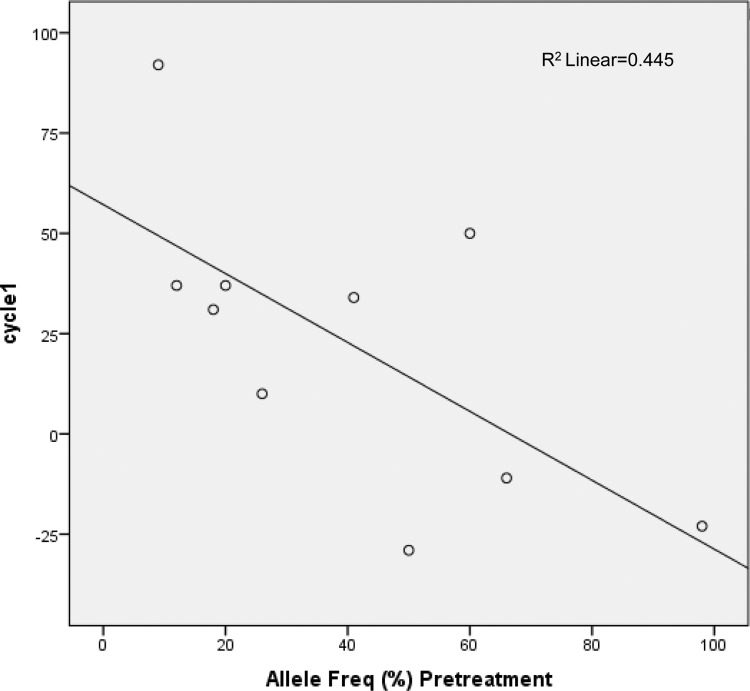
Correlation between percentage allele frequency and post-cycle 1 tumor response of at least 25%.

Cases from treatment arm B were significantly more likely to have change in mutation profile (MT→WT or WT→MT) than arm A, after adjustment for pre-chemotherapy mutation profile, presence of distant metastasis and ER status (adjusted odds ratio 12.00 [95% CI 1.11–129.87]). There was no significant association between baseline mutant samples harboring PIK3CA mutations (n = 8) versus those with other mutations (n = 2) in terms of tumor responses post-cycle 1 (p = 0.747) and overall tumor response (p = 0.098). However, baseline mutant tumors with <25% mutant allele frequency were more likely to achieve tumor reduction of ≥25% after cycle 1 chemotherapy (66.7% vs 33.3%, p = 0.035) than those with ≥25% mutant allele frequency. The same analysis (baseline tumors ≥25% vs <25% mutant allele frequency) for overall clinical response did not detect a significant difference between the mutation groups (P-value = 0.679). Upon Pearson correlation coefficient analysis, an inverse relationship was found between percentage mutant allele in the baseline tumour samples and post-cycle 1 chemotherapy absolute tumour reduction (R = -0.667, p = 0.035; Fig 3), suggesting that tumor containing a mutant allele was less likely to respond if it contained a high percentage of mutant allele.

**Fig 4:**
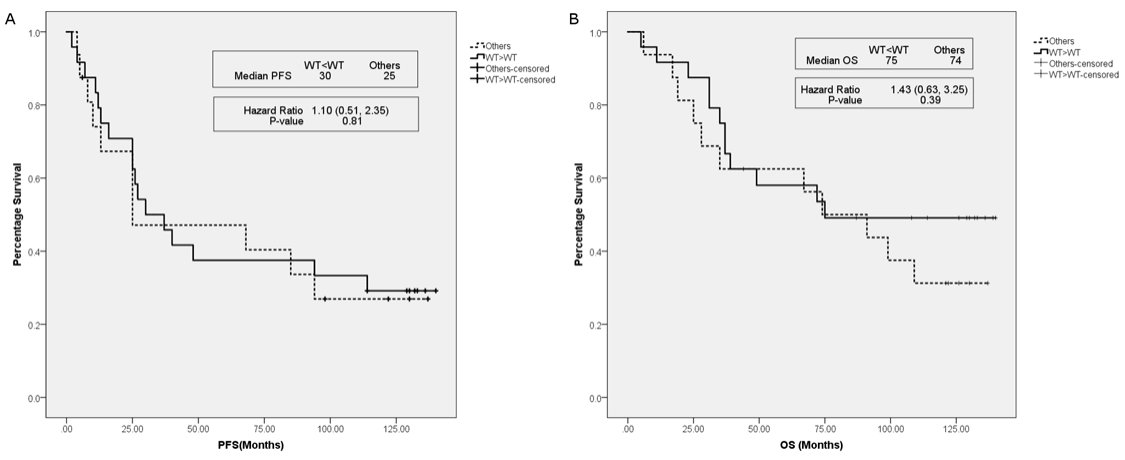
Progression-free and overall survival between the WT→WT subgroup versus combination of other mutation pattern subgroups. (A) Progression-free survival and (B) overall survival of the WT→WT subgroup and a combination of other mutation pattern subgroups (MT→MT, MT→WT, WT→MT). Bold line: WT→WT subgroup; broken line: combination of the other mutation subgroups MT→MT, MT→WT, WT→MT.

The PFS of WT→WT patients was not significantly different compared to the other 3 subgroups (hazard ratio {HR} 1.10 [95% CI 0.51–2.35]; *P* = 0.81) (Fig 4A). Similarly, no significant difference in OS was observed between WT→WT and the other 3 subgroups (HR 1.43 [95% CI 0.63–3.25]; *P* = 0.39) (Fig 4B). Multivariate analyses adjusting for T stage, presence or absence of metastases, ER and PgR status with respect to PFS and OS showed that the HR changed from 1.10 to 1.04 and 1.43 to 1.14 respectively, but the overall results remained unchanged.

## Discussion

To our knowledge, there have been limited studies that evaluated the influence of chemotherapy on mutational profiles in clinical breast cancer specimens. We examined the mutation profiles of 40 paired primary breast tumor biopsy samples pre- and post-chemotherapy 3 weeks apart after only one cycle of doxorubicin or docetaxel. The major findings were variation in mutation status between treatment-naive baseline samples and post-treatment samples in about a quarter of patients (11/40 or 28% WT→MT or MT→WT). Overall, the majority of baseline samples were WT at baseline (30/40; 75%), and 6/30 (20%) of these originally WT samples had new mutations detected in the corresponding post-treatment sample. Interestingly, all 6 samples were exposed to docetaxel rather than to doxorubicin. The majority of post-chemotherapy mutations that were uncovered after docetaxel exposure were less common mutations (*ABL*, *EGFR*, *MET*, *PDGFRA*), in contrast to the predominance of *PIK3CA* mutations observed in pre-treatment samples (8/10 baseline MT tumors harboured *PIK3CA* mutations). Half of the baseline MT tumors retained the same mutation after chemotherapy (MT→MT); all possessed *PIK3CA* mutations, and all but one were treated with doxorubicin. The remaining 5 MT tumors at baseline reverted to WT status after chemotherapy, with no clear pattern of prior drug exposure (doxorubicin or docetaxel); pre-treatment mutations in these tumors were also more diverse, in *PIK3CA* (n = 3), *EGFR* (n = 1), and *KIT* (n = 1).

Differing somatic mutations seen pre- and post-treatment as demonstrated in our study may be attributable to several key reasons. Firstly, data suggest that topological clonal heterogeneity within primary tumors exists, consisting of diverse mutations and a large number of subclonal and *de novo* mutations [9,10,11], and new clones with different mutational profiles may have developed spontaneously even in the few weeks that separated the pre- and post-chemotherapy biopsy. Additionally, certain rapidly proliferating MT or WT tumor cells may have a better response to chemotherapy, and this selective pressure from chemotherapy may lead to expansion of resistance clones [12]. Cells with different mutational profiles may have differing sensitivities to different chemotherapeutic agents; the observed loss of mutations after chemotherapy in our study may have been caused by increased sensitivity of certain mutation-containing clones to the treatment, hence primarily removing the mutant cells and consequently shifting the genetic evolutionary landscape in favour of the non-mutant subclones. These mutant alleles could still be present after chemotherapy but have fallen to such low frequencies that they were not detectable with our assay, and hence the tumors were considered to have changed from MT to WT. Similarly, certain wildtype clones may be very susceptible to chemotherapy, resulting in mutant clones becoming dominant in the post-treatment samples in some patients (MT→WT).

A noteworthy observation from our study is that all 6 originally WT tumor samples which acquired mutations post-treatment were exposed to docetaxel, instead of doxorubicin. One possible hypothesis for this observation is selective sensitivity of certain WT subclones to docetaxel thus enriching the post-treatment samples for non-WT cells, or pre-existence of docetaxel-resistant MT clones in low frequency in baseline samples which became more readily detectable after other subclones were eradicated with docetaxel.

The phenomenon of mutation variations post-treatment has been reported in other cancer types such as non-small cell lung cancer, where *EGFR* mutant to wild type changes were observed after chemotherapy and postulated to be related to intratumoral heterogeneity and differing chemosensitivity levels of mutant and wild-type cells[13]. It is unique that in our study, mutation changes were detected as early as 3 weeks after a single cycle of chemotherapy which could offer an early evaluation of the evolving mutational landscape of the tumor in response to drug treatment. The value of detecting potentially actionable mutations earlier on is in identifying new therapeutic targets which can provide insights on developing rational novel combinations or optimal sequencing of chemotherapy and targeted drugs to treat residual resistant disease. For example, the common *PIK3CA* hotspot mutations in our samples, namely *H1047R*, *E545K* and *E542K* are the targets of pan-PI3K inhibitors such as GDC-0941, BKM120, BYL719 and XL-147 which are being actively evaluated in combination with chemotherapy, endocrine therapy, and/or anti-HER2 agents in advanced breast cancers [14,15]. c-MET signalling inhibition has demonstrated activity in metastatic breast cancer using the drug cabozantinib, which targets *MET* mutations such as *Y1248H*, *D1246N* and *K1262R* [16,17,18]. *EGFR* mutations have been identified in triple-negative breast cancers in a small study highlighting the possible application of oral EGFR tyrosine kinase inhibition therapy in selected breast cancer patients [19]. Of interest is the *EGFR S768I* mutation identified in a baseline sample in our study that has demonstrated greater sensitivity to AEE788, an oral multi-receptor tyrosine kinase inhibitor, compared to erlotinib and gefitinib in an *in-vitro* study [20].

The analytical sensitivity of mutations for the Sequenom assay is typically around 10%[6,21]. Mutant allele frequencies observed did not show any consistent direction shift pre- and post-chemotherapy in the MT→MT group. Two out of the 5 paired samples in this MT→MT group exhibited a decrease in allele frequency post-chemotherapy. However, we believe it is risky to interpret a decreased allele frequency as representing a reduction in tumor clones with the mutation. Allele frequency can be affected by tumour content, hence a decreased allele frequency could simply reflect a lower tumor content in the sample. This issue is not specific to Sequenom, and is an issue for next generation sequencing and other genotyping assays performed on tissue samples with mixtures of tumor and normal tissue.

Interestingly, our analyses demonstrated a lower likelihood of tumor response if there was a high mutant allele frequency in a baseline mutant sample. Notably 8 out of our 10 baseline samples contained *PIK3CA* mutations. There has been some prospective data in the neoadjuvant setting reporting on the presence of *PIK3CA* mutations being associated with a poorer response and clinical outcome. This was observed in the neoadjuvant GeparQuattro, GeparQuinto, and GeparSixto studies[22], as well as the Neoadjuvant Lapatinib and/or Trastuzumab Treatment Optimization trial (NeoALTTO) study[23], where patients carrying a *PIK3CA* mutation had decreased pathologic complete responses compared with those who had wild-type *PIK3CA*. Although our sample size is small, our findings appear to be concordant with these reports.

In our study, we used core biopsy to obtain tumor specimens. Single core biopsy samples with limited tissue might not represent the complete genomic diversity of the tumor, and minute proportions of mutant clones may be overlooked due to sampling bias. In particular, all the patients in our study had large clinical T3 or T4 tumors, and a single core biopsy in such large tumors will inevitably result in sampling bias. Interestingly in our study, only one mutation was identified per biopsy which highlights the limitation of single biopsies which contain small amount of material which could lead to under-estimation of the tumor mutational landscape. However a core biopsy is still logistically most feasible in the clinic, although performing multiple baseline core biopsies or perhaps even an excisional biopsy, or repeating tumor biopsies for the primary and recurrent lesions during the course of treatment may be solutions to provide a better reflection of the entire tumor genomic landscape to guide therapy.

We recognise that our study has several important limitations. Firstly, our sample size is small, although it was reassuring that the frequencies and types of mutations detected in our samples at baseline were comparable to the Catalogue of Somatic Mutations in Cancer (COSMIC) database (*PIK3CA*, 8/40, 20%; *EGFR*, 1/40, 2.5%; *KIT*, 1/40, 2.5%) and other studies [24,25,26]. Secondly, the OncoCarta panel interrogates a finite number of mutations; for example, although *TP53* is the second most frequently mutated gene after the *PI3KCA* proto-oncogene occurring in approximately 23% of breast cancer samples [27], it was not included in our assay panel. Furthermore, while Sequenom assays have high sensitivity with as little as 1ng of DNA, mutations are only detectable if present in at least 5–10% of the total DNA [6]. This assay is thus far less sensitive compared with current technology such as deep sequencing which albeit cost prohibitive and difficult to apply routinely, enhances mutation detection rate in low purity, highly polyclonal tumors.

Our findings highlight that tumor mutational profiles can change as quickly as after one cycle of chemotherapy in breast cancer, underscoring the problem of intra-tumoral heterogeneity and the evolving tumor as potential major barriers to using a single predictive biomarker test and at a single timepoint to guide treatment. Potential strategies to overcome the hurdle of tumor heterogeneity include tissue collection at multiple tumor sites, routine tissue collection at relapse, profiling of circulating tumor cells, or more recently measurement of circulating free tumor DNA as surrogates, which could allow repeated and more comprehensive profiling of heterogeneity and an improved understanding of potential resistance mechanisms [28,29]. Emerging technologies such as single-cell genome and exome sequencing or deep sequencing of tumors may further assist in characterizing intra-tumoral heterogeneity [28].

## Conclusion

In conclusion, our study highlighted differences in mutation status in pre- and post-chemotherapy breast tumours. This phenomenon may be due to pre-existing intra-tumoral heterogeneity detected by sampling bias, cytotoxic therapy applying selective pressure and leading to an enhanced tumor evolutionary rate, or direct drug-induced genetic aberrations. Mutations detected pre- and especially early on post-chemotherapy exposure could guide us in the identification and development of additional druggable targets to enhance therapeutic response.

## Supporting Information

S1 CONSORT Checklist (DOC)

S1 Protocol Study Protocol. (PDF)

S1 Table OncoCarta v1.0: 238 mutations from 19 oncogenes. (PDF)
