# Supplementary material for: High-Throughput Mutation Profiling Changes before and 3 Weeks after Chemotherapy in Newly Diagnosed Breast Cancer Patients
Source: PLoS One. 2015 Dec 2;10(12):e0142466. doi: 10.1371/journal.pone.0142466 (PMC4667877; doi:10.1371/journal.pone.0142466)
Supplement: S1 Protocol — (PDF) [file pone.0142466.s002.pdf]

② 11/11/09

6|00|502 - ①

Department of Haematology-Oncology
National University Hospital

Gene Expression Profiles of Breast Cancer Treated with Sequential Adriamycin and Docetaxel in relation to Pharmacokinetics and Tumor Response
Protocol No.: HO B17/02

Principal Investigator:
National University Hospital: Dr Lee Soo Chin[1]
Tel: (65) 6772-4621
Fax: (65) 6777-5545
email: LeeSC@nuh.com.sg

Co-Investigators:
National University Hospital: Dr Goh Boon Cher[1]
Dr Lim Siew Eng[1]
Dr Philip Iau Tsau Choong[2]
Professor John Wong Eu-Li[1]
Dr Thomas Putti[3]
Dr Yong Wei Peng[1]
Dr Elaine Lim Hsuen[1]
Dr Wong Seng Weng[1]
Dr Tan Sing Huang[1]

[1]: Department of Haematology-Oncology, NUH
[2]: Department of Surgery, NUH
[3]. Department of Pathology, NUH

Genome Institute of Singapore: Professor Edison Liu Tak Bun

Johns Hopkins-NUH International Medical Centre: Professor Alex Chang Yuang-Chi

Study Site
Department of Haematology-Oncology
National University Hospital
5 Lower Kent Ridge Road
Singapore 119074
Tel: (65) 6772-4140
Fax: (65) 6777-5545

Research Nurses: Ms Norita bte Sukri
Tel: (65) 6772-4625
Fax: (65) 6872-3137
email: norita@nuh.com.sg

Supported by: Aventis

Table of contents

1. **Introduction.**
    1.1. Molecular biology and cancer treatment
    1.2. Tumor genetic profile as a predictor of treatment response
    1.3. Patient genetic profile as a predictor of drug toxicity
    1.4. Singapore as a major contributor to Asian genetics research
    1.5. Breast cancer as a model to study cancer gene expression

2. **Aims of Research Proposal**
    2.1. Hypothesis
    2.2. Primary objective
    2.3. Secondary objectives

3. **Investigational Plan**
    3.1. Summary of Study Design
    3.2. Study schema
    3.3. Investigator information
    3.4. Study population
        3.4.1. Criteria for enrollment
            3.4.1.1. Inclusion criteria
            3.4.1.2. Exclusion criteria
        3.4.2. Patient Assignment
    3.5. Dosage and Administration
        3.5.1. Material and Supplies
        3.5.2. Dosage Administration
        3.5.3. Treatment plan
        3.5.4. Premedications
    3.6. Dose modifications
        3.6.1. Dose adjustments or delays for subsequent cycles
        3.6.2. Haematologic Toxicity
        3.6.3. Non-haematologic toxicities
            3.6.3.1. Liver toxicity
            3.6.3.2. Peripheral neuropathy
            3.6.3.3. Myalgia, Arthalgia, Asthenia, Fatigue
            3.6.3.4. Hypersensitivity reaction
            3.6.3.5. Cardiac toxicity
    3.7. Concomitant treatment
        3.7.1. Colony stimulating factors
    3.8. Efficacy evaluation
        3.8.1. Baseline Assessments
        3.8.2. Timing of subsequent tumor assessments
        3.8.3. Efficacy criteria for tumor response
            3.8.3.1. Disease status and measurability
            3.8.3.2. Objective Response Status
            3.8.3.3. Definition of Efficacy Measures

3.9. Safety
3.9.1. Safety Measures
3.9.2. Clinical Adverse Events
3.9.3. Serious Adverse Events (SAE)
3.9.3.1. Definition of SAE
3.9.3.2. Reporting of SAE
3.9.3.3. Follow-up of SAE
3.10. Discontinuation
3.11. Clinical laboratory tests and procedures
3.11.1. Pre-study
3.11.2. During the study
3.11.2.1. Efficacy assessment
3.11.2.2. Safety assessment
3.11.3. Laboratory tests and results
3.11.4. Post-study follow-up
3.11.4.1. Efficacy
3.11.4.2. Safety

4. Sample size and data analysis methods
4.1. Sample size
4.2. Data to be analyzed

5. Clinical Pharmacy

6. Special Tests
6.1. Pharmacokinetics studies
6.2. Genotyping studies
6.3. Gene expression studies and cluster analysis
6.4. Gene expression studies of peripheral mononuclear cells

7. Study monitoring and data collection
7.1. Data collection
7.2. Data management
7.3. Statistical analysis

8. Informed consent, ethical review, and regulatory considerations
8.1. Informed consent
8.2. Patient information
8.3. Ethical review
8.4. Regulatory considerations

9. References

10. Appendix
10.1. AJCC staging criteria
10.2. Karnofsky performance status
10.3. Patient information sheet & consent Form
10.4. Evaluation and visit schedule

1. Introduction

1.1. Molecular biology and cancer treatment

Advances in the field of genomic and proteomic research has brought a new frontier to the understanding of molecular events relating to cancer susceptibility, cancer development, angiogenesis and metastasis, and treatment response. The imminent completion of the Human Genome Project will make available a blueprint to scientists for gene discovery.[1] The next step of advancement would be to characterize the functions, forms, and regulation of these genes with respect to disease states and therapy. The integration of genomics with proteomics would elucidate crucial biological pathways.[2] In the field of oncology, such studies could uncover important molecular targets in the development and maintenance of the malignant state, as well as elucidate mechanisms of resistance to drug and radiation therapy.[3] Such molecular knowledge can be applied clinically to refine cancer treatment. As an example, the genetic profiles of the tumor and the patient may be used to select the most appropriate type and dose of chemotherapeutic agent to achieve the best tumor efficacy while minimizing toxicity to normal tissues.

1.2. Tumor genetic profile as a predictor of treatment response

Conventional chemotherapy to treat cancer lacks sophistication in its inability to accurately predict treatment efficacy and drug toxicity for the individual patient. For decades, morphologic features have been used to predict treatment response and prognosis. As an example, different cancer types respond differently to chemotherapy: adriamycin is active in breast,[4] but not in colorectal cancer; all-trans retinoic acid has remarkable activity in the promyelocytic form of acute leukaemia, but not in other subtypes.[5] More recently, the use of molecular markers to predict treatment response and prognosis in specific tumor types has been incorporated into clinical practice. In breast cancer, the presence of estrogen or progesterone receptors is the most powerful predictor of tamoxifen response,[6] while c-erb-B2 over-expressing tumors demonstrate relative resistance to conventional non-anthracycline containing chemotherapy, and may be more appropriately treated with dose-intense anthracycline-containing regimens.[7] In non-small cell lung cancer, the presence of β-tubulin mutations results in tumor resistance to anti-tubulin agents such as paclitaxel.[8] In gliomas, high expression of O6-methyl guanine-DNA methyltransferase (*MGMT*) results in poorer survival following carmustine treatment, while silencing of the gene by methylation leads to better overall survival.[9] Thus, treatment may potentially be modified based on the molecular profile of the tumor to achieve more consistent and predictable responses. An excellent example of the application of molecular knowledge in cancer therapeutics is the recent success of glivec, a specific inhibitor of the BCR-ABL tyrosine kinase in the treatment of chronic myeloid leukaemia.[10, 11]

Up to recently, isolated molecular markers have been used to predict tumor response. Such a strategy is fraught with difficulty, since tumor response is often a function of complex interactions of multiple genes and pathways. The simultaneous analysis of thousands of genes has now been made possible by micoarray technology.[12] Such technology has the ability to identify important genes in relation to particular functions, and has the further potential to elucidate interactive relationships between genes.[13] The ability to study mRNA arrays using fine needle samples has further facilitated these studies as samples can be taken safely and repeatedly.[14, 15]

More recently, protein expression profiles in tumors have been studied using ProteinChip technologies with surface enhanced laser desorption / ionization – mass spectrometry (SELDI-MS).[16, 17] These studies are complementary to gene expression studies, and together could yield valuable data providing insight into important genes and pathways in tumorigensis and drug resistance.

1.3. Patient genetic profile as a predictor of drug toxicity

The 'optimal' doses of conventional chemotherapy have largely been determined from maximally tolerable doses (MTD) derived from the study of a limited cohort of patients based on toxicity. Unfortunately, there is significant inter-individual variability in pharmacokinetics resulting in a wide range of side effects including fatal toxicities, despite corrections for weight, height, renal and hepatic function.[18-20] Even the commonly used body surface area to individualize chemotherapy dosing has recently been reported to correlate poorly with drug clearance.[21] Inter-individual differences in drug efficacy and toxicity are in part related to genetic polymorphisms in drug metabolizing enzymes (DMEs), transporters and receptors.[22] The study of these genetic polymorphisms in relation to drug pharmacokinetics (PK) and pharmacodynamics (PD) can potentially lead to more rational therapeutics that reduces risks of severe toxicity. Several examples currently exist where subjects carrying certain alleles suffer from a lack of efficacy or increased toxicity to drugs commonly used in different fields of medicine. The *CYP2C9*2* and *CYP2C9*3* allele have been associated with retarded elimination of S-warfarin, lower maintenance doses and more frequent bleeding episodes.[23] Homozygotes for the *C677T* polymorphism in the 5,10-methylenetetrahydrofolate reductase (*MTHFR*) gene had lower MTHFR activity and experienced increased methotrexate toxicity,[24] while genetic polymorphisms of thiopurine methyl transferase were reported to be an important determinant of mercaptopurine toxicity.[25] Individuals homozygous for the *C3435T* polymorphism of the multidrug resistance (*MDR*)-*1* gene have significantly lower duodenal MDR-1 expression and experienced higher plasma digoxin levels compared to normal homozygotes.[26] A single base-pair substitution in the dihydropyrimidine dehydrogenase (*DPD*) gene resulted in complete deficiency of the DPD enzyme and potentially lethal toxicity to 5-fluorouracil.[27] The clinical application of such knowledge has the potential to reduce treatment related toxicity, while maintaining efficacy.

Gene expression changes in the peripheral blood mononuclear cells could be a good surrogate marker to predict toxicity following chemotherapy as it is a readily accessible source of material. The gene sets or profiles obtained could be diagnostic for certain forms of toxicity. This principle of generating transcriptional fingerprints in relation to chemical perturbations or chemotherapy has been demonstrated *in vitro*[28, 29], in acute myeloid leukemia[30] and in acute lymphocytic leukemia[31, 32] among others. The feasibility of obtaining gene expression patterns from mononuclear cells in the peripheral blood has been demonstrated in normal healthy volunteers[33] as well as in patients undergoing chemotherapy.[34]

1.4. Singapore as a major contributor to Asian genetics research

Genetic polymorphisms demonstrate ethno-geographic differences.[35] For example, the N-acetyl-transferase-2 gene is polymorphic, with Orientals being generally "fast acetylators" and Caucasians "slow acetylators".[36] Similar ethnic differences in genetic polymorphisms have also been observed for *Cyp2C19*,[37-39] *Cyp2D6*,[37, 40, 41] *UGT1A1*,[42, 43] and the *MDR-1* gene.[44] While extensive genotypic data has been generated for Western patients, similar information has been lacking in Asians. Much of the current existing information have been based on Asian subjects in the West.[43, 45, 46] These data often suffer from small sample size,

and may not represent information derived from a homogeneous group, since subjects from different Asian ethnic groups are lumped together to form a heterogeneous group and called the 'Asian' data.[47-49] There are significant phenotypic differences between these ethnic groups even within Asia,[50] which may in turn reflect differences in genotypic make-up.[38, 41] Thus, it is important that a more homogeneous dataset is generated from within Asia. Singapore is ideal for such studies and could be a major contributor to Asian pharmacogenetics data. The presence of three major resident ethnic groups in our population, namely Chinese, Malay and Indian, offers the added opportunity to study and compare drug handling phenotype (PK and PD) and genotype between distinct ethnic groups in an attempt to address possible racial differences in drug handling and tumor response.

1.5. Breast cancer as a model to study cancer gene expression

We plan to study the genetic profiles of tumor and patient in relation to chemotherapy with the ultimate goal of using the information to refine cancer treatment for each individual. We have selected breast cancer as the model, and will specifically study stage II to IV breast cancer patients with measurable primary breast tumor and who are receiving chemotherapy as first-line treatment. This is an ideal model as breast cancer is common in Singapore, is chemosensitive, and primary breast tumor is easily amenable to repeated sampling for genetic studies. Pre-operative chemotherapy in early stage and locally advanced breast cancer is established treatment that results in similar survival outcome as conventional post-operative treatment, with the added advantages of improved drug delivery through better blood supply, tumor down-sizing to facilitate breast conservation, and the opportunity to assess tumor chemosensitivity.[51] While combination chemotherapy comprising of two or three agents is conventionally given in such a setting because of superior response rate, it has no proven survival benefit over single agents administered sequentially. In this study, we adopt the strategy of using two of the most active agents in breast cancer, adriamycin and docetaxel, administered as single agents in a sequential fashion. Such a strategy allows tumor gene expression profiles and drug pharmacokinetics to be studied without the confounding effects of another drug. Sequential single agents also have the potential advantages of allowing higher doses of each drug to be administered, resulting in maximal cell kill and avoiding antagonistic interactions between drugs. We have designed a phase II study where patients could be randomized to one of two different sequences of adriamycin and docetaxel. This provides the opportunity to study differences in tumor response and gene expression changes in relation to different sequences of drugs. Pharmacokinetics and genotyping studies will be carried out and correlated with treatment response and toxicity. Specific genes to be studied include *CYP3A5* and *MDR1* that affect docetaxel clearance, and glutathione-S-transferase (*GST*) that affects adriamycin metabolism.

This study would make available collaborative data that allows for multi-dimensional analysis to identify (1) "cluster gene patterns" that may predict good clinical response to chemotherapy, and (2) differential gene expressions in response to different cytotoxic agents from which genes of drug resistance may be sieved out. Crucial genes that affect tumor response and resistance may be further investigated comprehensively with such phenotype-genotype linkage based on microarrays. Our study will comprehensively study the relationship of genotype of multiple DMEs on more than one drug in the same patient. To our knowledge, this has not been done previously. The results of this study will be an important step forward in fulfilling the promise of individualized dosing based on genetic information.

2. Aims of Research Proposal

2.1. Hypothesis

We hypothesize that changes in tumor gene expression profiles vary in response to different sequences and types of chemotherapy, and that gene expression changes will correlate with tumor response. We are also looking to correlate drug pharmacokinetics and treatment toxicity with genotype of drug metabolizing enzymes and tranporters.

2.2. Primary Objective

1. Evaluate the impact of adriamycin and docetaxel on tumor gene expression profiles.
2. Correlate overall tumor response with tumor gene expression profiles.

2.3. Secondary objectives

To correlate adriamycin and docetaxel pharmacokinetics with:

1. Genetic polymorphisms of *MDR-1*, *Cyp3A* and *GSTs*.
2. Drug toxicity and tumor response.
3. Peripheral mononuclear cell gene expression profiles

3. Investigational Plan

3. 1 Summary of study design

This is a single-centre, open-label, randomized phase II study of two different schedules of sequential adriamycin and docetaxel in breast cancer.

A total of one hundred patients with measurable disease will be enrolled and randomly assigned to each of two study arms:

Arm A: A→T→ A→T→ A→T

Arm B: T→A→T→ A→T→ A

where 'A' represents adriamycin 75mg/m^2 q 3 weeks and 'T' represents docetaxel 75mg/m^2 q3 weeks

Tumor core biopsy will be performed before treatment, following the first cycle of adriamycin, following the first cycle of docetaxel, and at study withdrawal or study completion for a total of four tumor cores for gene expression studies. The final biopsy may be obtained at surgery if the patient is scheduled for lumpectomy or mastectomy. The tumor cores will be stored in liquid nitrogen for subsequent RNA extraction and gene expression studies.

Blood sampling will be carried out for pharmacokinetic studies for adriamycin and docetaxel during the first cycle of each chemotherapeutic agent.

10ml blood will be taken from each participant prior to the start of treatment for genotyping studies.

3.2 Study schema

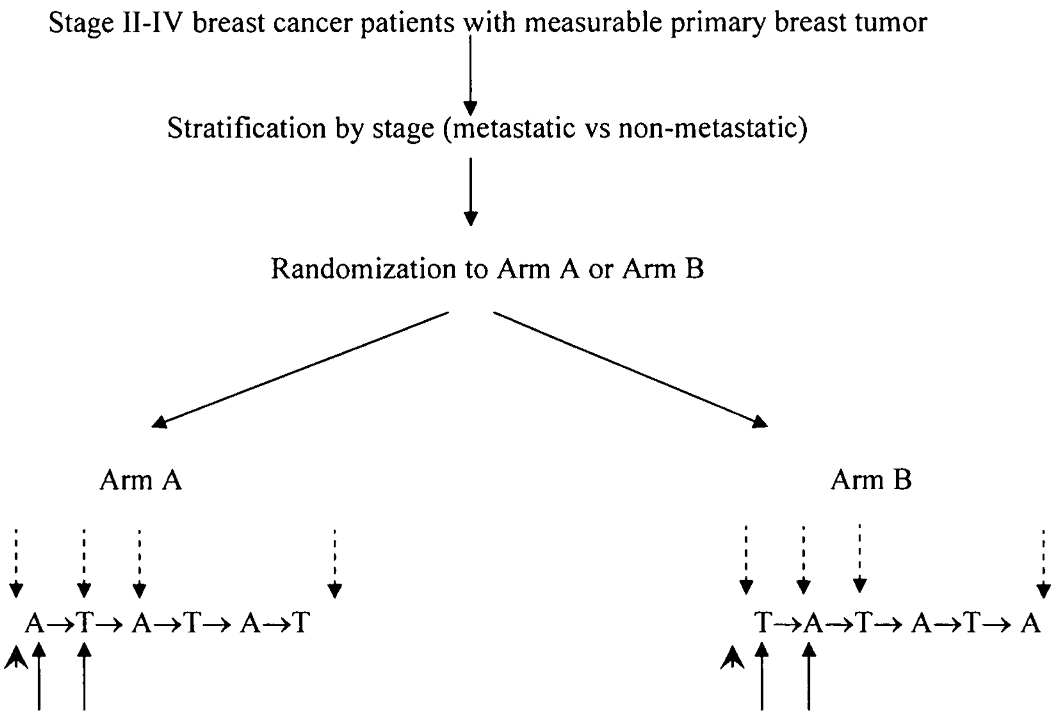

A: Adriamycin 75mg/m^2 q 3 weeks
T: Docetaxel 75mg/m^2 q 3 weeks

Core biopsy of breast tumor before treatment, after cycle 1 of adriamycin, after cycle 1 of docetaxel, and at study withdrawal or study completion for a total of 4 core biopsies

Pharmacokinetics studies during cycle 1 of each chemotherapy drug

Blood for patient **genotype**

Data analysis

1. Comparison of acute genetic and protein expression changes in tumor in response to the two different schedules of chemotherapy (ie Arm A versus Arm B).
2. Correlation between clinical response (good clinical response, stable disease and progressive disease) and tumor gene and protein_expression changes.
3. Correlation between drug pharmacokinetics and treatment toxicity/tumor response.
4. Correlation between patient genotype and drug pharmacokinetics.

3.3 Investigator Information

The names, titles, and institutions of the investigators are listed in the Contacts for Protocol. If investigators are added after the ethical review board and/or the local regulatory agency have approved the study, these additions will not be considered changes to the protocol, but the Contacts for Protocol will be updated to provide this information.

3.4. Study Population

3.4.1. Criteria for enrollment

3.4.1.1. Inclusion criteria

Patients may be included in the study only if they meet **all** of the following criteria:

- Female, age ≥ 18 years.
- Histologic or cytologic diagnosis of breast carcinoma.
- Stage II to IV breast cancer with measurable primary breast tumor, defined as palpable tumor with both diameters 2.0cm or greater as measured by caliper.
- Patients must not have received prior chemotherapy or hormonal therapy for the treatment of breast cancer.
- Karnofsky performance status of 70 or higher.
- Estimated life expectancy of at least 12 weeks.
- Adequate organ function including the following:
  - Bone marrow:
    - White blood cells (WBC) ≥ 3.5 x 10^9/L
    - Absolute neutrophil (segmented and bands) count (ANC) ≥ 1.5 x 10^9/L
    - Platelets ≥ 100 x 10^9/L
    - Haemoglobin ≥ 9g/dL
  - Hepatic:
    - Bilirubin ≤ 1.5 x upper limit of normal (ULN),
    - ALT or AST ≤ 2.5x ULN, (or ≥5 X with liver metastases)
    - Alkaline phosphatase ≤ 2.5x ULN.
  - Renal:
    - creatinine ≤ 1.5x ULN
  - Cardiac:
    - Adequate cardiac function
- Signed informed consent from patient or legal representative.
- Patients with reproductive potential must use an approved contraceptive method if appropriate (eg, intrauterine device, birth control pills, or barrier device) during and for three months after the study. Females with childbearing potential must have a negative serum pregnancy test within 7 days prior to study enrollment.

3.4.1.2. Exclusion criteria

Patients will be excluded from the study for **any** of the following reasons:

- Prior treatment for locally advanced or metastatic breast cancer.
- Treatment within the last 30 days with any investigational drug.
- Concurrent administration of any other tumor therapy, including cytotoxic chemotherapy, hormonal therapy, and immunotherapy.
- Active infection that in the opinion of the investigator would compromise the patient's ability to tolerate therapy.
- Pregnancy.

- Breast feeding.
- Serious concomitant disorders that would compromise the safety of the patient or compromise the patient's ability to complete the study, at the discretion of the investigator.
- Poorly controlled diabetes mellitus.
- Second primary malignancy that is clinically detectable at the time of consideration for study enrollment.
- Symptomatic brain metastasis.
- History of significant neurological or mental disorder, including seizures or dementia.
- Peripheral neuropathy of ≥ CTC grade 2.
- History of hypersensitivity to drugs formulated in Tween 80, the vehicle used for commercial docetaxel formulations.

3.4.2. Patient Assignment
Eligible patients will be stratified by stage (metastatic or non-metastatic) at a 3:7 ratio. Accordingly, we will recruit a total of 70 non-metastatic and 30 metastatic patients, to be randomized in equal proportion to either Arm A or Arm B.

Patients will be entered into the trial by a telephone call to the Cancer Therapeutics Research Group (CTRG), Singapore (+65 6772-4628) between 0830 to 1730 hours from Monday to Friday, and between 0830 to 1230 hours on Saturday (Singapore time), or by Fax (+65 6872-3137) at any time, stating that the patient is to be entered into the trial. Confirmation fax will be sent to the investigator.

Written informed consent for entry into the study will be obtained prior to randomisation. All eligibility criteria and consent form will be checked before treatment is allocated.

3.5. Dosage and Administration
3.5.1. Material and Supplies
Adriamycin and docetaxel will be obtained locally from the manufacturer. Storage conditions for unopened vials, reconstitution, and storage conditions for the reconstituted solution will follow the manufacturer's recommendations. Docetaxel solutions should be prepared and stored in glass, polypropylene or polyolefin containers. Non-PVC containing and polyethylene-lined administration sets should be used.

3.5.2. Dosage Administration
A cycle is defined as an interval of 21 days. A delay of cycle due to holidays, weekends or bad weather will be permitted and not counted as a protocol violation.

The actual doses of adriamycin and docetaxel administered to subjects will be determined by calculating the body surface area at the beginning of the cycle. A ± 5% variance in the calculated total dose will be allowed for ease of dose administration.

3.5.3. Treatment plan

Arm A: A→T→ A→T→ A→T

Arm B: T→A→T→ A→T→ A

where 'A' represents adriamycin 75mg/m^2 q 3 weeks and 'T' represents docetaxel 75mg/m^2 q3 weeks

3.5.4. Premedications

Docetaxel

Patients receiving docetaxel should receive routine pre-medications with oral dexamethasone 8mg twice daily for 3 days, starting 1 day prior to each docetaxel administration. Anti-emetics are prescribed according to the investigator's preference.

Adriamycin

Anti-emetics are prescribed according to the investigator's preference.

3.6. Dose modifications

3.6.1. Dose adjustments or delays for subsequent cycles

Any patient who requires a dose reduction based on nadir haematological toxicity or non-haematological toxicity will continue to receive a reduced dose for the remainder of the study. Any patient with a prior dose reduction who experiences a toxicity that would cause a third dose reduction must be discontinued from the study therapy. Treatment may be delayed for up to 42 days from day 1 of the current cycle to allow a patient sufficient time to recover from study drug-related toxicity. A patient who cannot start the next cycle for 42 days from day 1 of the current cycle must be discontinued from study therapy unless the Principal Investigator approves continuation.

3.6.2. Haematologic Toxicity

Dose adjustments at the start of a subsequent course of therapy will be based on nadir platelet and neutrophil counts from the preceding cycle of therapy. ANC must be ≥ 1.5x 10^9/L prior to the start of a new cycle. Treatment may be delayed for up to 42 days from day 1 of the current cycle to allow proper time for recovery. Upon recovery, if treatment is resumed, the dose of study drugs must be adjusted according to the guidelines in Table 1.

Table 1: Dose adjustments for adriamycin and docetaxel based on nadir hematology values for preceding cycles		
Platelets ($x10^9/L$)	ANC ($x10^9/L$)	Percent of day 1 dose in previous cycle
≥ 25	≥ 1.0	100%
≥ 25	0.5 – 0.99 if not associated with documented infection or fever	100%
≥ 25	<0.5 lasting for <7 days and not associated with documented infection or fever	100%
≥25	0.5 – 0.99 if associated with documented infection or fever	80%
≥ 25	<0.5 lasting for ≥7 days or associated with documented infection or fever	80%
25-49.9 if associated with bleeding	Any	80%
<25	Any	80%

3.6.3. Non-haematologic toxicities

If dose adjustment is required for non-hematological toxicity, the patient will continue to receive the reduced dose for the remainder of the study, unless otherwise specified.

3.6.3.1. Liver toxicity

If at the start of the next treatment cycle serum bilirubin is elevated to >1.5x ULN, or AST/ALT are elevated to >2.5x ULN in patients without liver metastasis, or >5.0x ULN in patients with liver metastasis, respectively, the cycle should not begin until serum bilirubin/ALT/AST have returned to below these levels. If the laboratory values do not return to these limits within 42 days from day 1 of the current cycle, the patient should be discontinued from study therapy. In the event of CTC grade 3 or 4 liver toxicity (ALT/AST/serum bilirubin), subsequent administration of the particular chemotherapy drug should be discontinued. Administration of the alternative chemotherapy drug is allowed provided that levels have recovered to eligibility levels.

3.6.3.2. Peripheral neuropathy

In the event of CTC grade 2 peripheral neuropathy, subsequent doses of docetaxel should be reduced by 20% in all administrations and cycles. In the event of CTC grade 3 or 4 peripheral neuropathy, study treatment should be discontinued.

3.6.3.3. Myalgia, Arthralgia, Asthenia, Fatigue

In the event of CTC grade 2 mylagia, arthalgia, asthenia or fatigue lasting for more than 7 days, subsequent doses of docetaxel should be reduced by 20% in all administrations and cycles. In the event of CTC grade 3 or 4 myalgia, arthralgia, asthenia or fatigue, study treatment should be discontinued.

3.6.3.4. Hypersensitivity reaction

If in spite of administration of proper pre-medication, a hypersensitivity reaction occurs, the specific treatment that is medically indicated for given symptoms should be instituted immediately. Continuation or discontinuation of study treatment will be at the discretion of the investigator, and will depend on the severity of the hypersensitivity reaction, tumor response, and the medical judgment by the investigator if it is in the best interest of the patient to continue or to discontinue study treatment. If study treatment will continue, the subsequent doses of docetaxel should not be reduced.

Table 2: Management of hypersensitivity reactions for patients receiving docetaxel

Mild symptoms (CTC grade 1): Localized cutaneous reaction such as mild, transient pruritus, flushing or rash, drug fever <38°C	Consider decreasing the rate of infusion until recovery of symptoms; stay at bedside. Upon resolution of symptoms, complete infusion of docetaxel at the initial planned rate.
Moderate symptoms (CTC grade 2): Urticaria, drug fever ≥38°C, asymptomatic bronchospasm	Stop docetaxel infusion, give IV dexamethasone 10mg and/or IV diphenhydramine 50mg. Resume taxane infusion after recovery of symptoms
Severe symptoms (CTC grade 3): Bronchospasm, generalized urticaria, drop in systolic blood pressure to ≤80mmHg, angioedema	Stop docetaxel infusion, give IV dexamethasone 10mg and/or diphenhydramine 50mg and/or epinephrine, as appropriate. DISCONTINUE DOCETAXEL THERAPY.
Anaphylactic reaction (CTC grade 4)	Stop docetaxel infusion, give IV dexamethasone +/- diphenhydramine 50mg +/- epinephrine +/- general measures for treatment of patients with anaphylactic shock, as appropriate. DISCONTINUE DOCETAXEL THERAPY

3.6.3.5. Cardiac toxicity

Adriamycin should be discontinued if LVEF drops by 20% from baseline or drops to ≤30%.

3.7. Concomitant therapy

Patients are allowed to receive full supportive therapies concomitantly during the study. No other anti-tumor therapy (chemotherapy, immunotherapy, hormonal cancer therapy, surgery for cancer, radiotherapy for cancer) or experimental medications will be permitted while the patients are receiving study therapy. Any disease progression requiring other forms of specific anti-tumor therapy will be cause for early discontinuation of study therapy.

3.7.1. Colony stimulating factors

Routine use of colony stimulating factors is not permitted in this study. Patients should not receive prophylactic granulocyte colony stimulating factor (G-CSF) in any cycle. G-CSFs for subsequent cycles should only be considered for patients who have ANC <0.5 x 10^9/L, neutropenic fever, or documented infections while neutropenic. G-CSF must be discontinued at least 24 hours prior to the start of the next cycle of chemotherapy.

3.8. Efficacy Evaluations

3.8.1. Baseline Assessments

Clinical Assessments: Within 2 weeks prior to enrollment, physical examination will be performed for measurement of palpable tumor lesions by caliper or ruler.

Radiological Assessments: Within 4 weeks prior to enrollment, baseline radiological imaging studies for tumor assessment as appropriate will be performed on each patient. A baseline plain chest X-ray is mandatory. All other imaging studies will be at the discretion of the investigator.

3.8.2. Timing of subsequent tumor assessments

The same clinical evaluation and radiological imaging methods used at baseline must be used consistently for subsequent tumor assessments.

Clinical Assessment: To be repeated every 3 weeks prior to the start of every treatment cycle.

Radiological Assessment: To be repeated approximately every 6 weeks as appropriate, prior to the start of every other treatment cycle.

If the patient's disease has responded to therapy (either using clinical or radiological assessment), the response should be confirmed after at least 3 weeks from the first documentation of response.

3.8.3. Efficacy criteria for tumor response

3.8.3.1. Disease status and measurability

Measurable disease: Bi-dimensionally measurable lesions with clearly defined margins and two perpendicular diameters that are clearly measurable by any of the following:

- Palpation, with both diameters 2.0cm or greater as measured by caliper.
- Inspection, with both diameters 2.0cm or greater as measured by caliper.
- Chest X-ray, with at least one diameter 1.0cm or greater.
- Computerized tomography (CT), with both diameters greater than 1.0cm.

Evaluable disease: Uni-dimensionally measurable lesions with one clearly measurable diameter by any of the following:

- Chest X-ray, with one measurable diameter of 2.0cm or greater.
- Computerized tomography (CT) with one measurable diameter of 2.0cm or greater.

Non-evaluable disease: Ascites, pleural effusion, bone metastases, diffuse or confluent skin metastases without clear margins, disease documented by indirect evidence only (eg, by laboratory values).

All documented lesions are to be followed. If an organ has multiple measurable lesions, choose three to be followed before the patient is entered on study. The remaining measurable lesions in that organ will be documented and considered evaluable for the purpose of objective status determination.

3.8.3.2. Objective Response Status

Objective response will be recorded at each evaluation.

Complete response (CR): Complete disappearance of all measurable and evaluable disease. No new lesions. No disease-related symptoms. No evidence of non-evaluable disease, including normalization of abnormal laboratory values secondary to metastatic breast cancer.

Minimal residual disease (MRD)[52, 53]: Residual palpable irregularity at the site of the primary tumor that is too small to be measured, representing an almost CR to treatment

Partial response (PR): Defined as ≥50% reduction relative to baseline in the sum of products of perpendicular diameters of all measurable lesions. No progression of evaluable disease. No new lesions.

Stable disease (SD): Does not qualify for CR, PR, or progressive disease.

Progressive disease (PD):

- ≥25% increase in the sum of products of bi-dimensionally measurable lesions *OR*
- obvious worsening of any evaluable disease *OR*
- reappearance of any lesion which had disappeared *OR*
- appearance of any new lesion/site *OR*
- failure to return for evaluation due to death or deteriorating condition (unless clearly unrelated to the cancer).

Worsening of existing non-evaluable disease does not constitute progression.

In patients with pre-existing bone metastases, the appearance of any new bone lesion as documented by bone scan or skeletal X-ray constitutes progression.

Exception: Lesions that appear to increase in size due to the presence of necrotic tissues will not be considered to have progressed.

Unknown: Progression has not been documented and one or more measurable or evaluable sites have not been assessed.

Best response: Two objective status determinations of CR, PR or SD before disease progression are required for a best response of CR, PR or SD respectively. Patients with an objective status of PD on or before the second on-study treatment evaluation will have a best response of PD. Best response is unknown if the patient does not qualify for a best response of PD and if all objectives statuses after the first determination and before progression are unknown. For CR and PR, responses must be confirmed by a second assessment 3 to 4 weeks (minimum 21 days) after the first documentation of response, using the same method of measurement as at baseline.

Good clinical response[53]: Defined as CR, MRD or PR. This has been shown to be a valid surrogate marker for survival.[53]

3.8.3.3. Definition of Efficacy Measures

A tumor responder is defined as any patient exhibiting a best study response of CR, MRD or PR (based on palpation and/or imaging).

Among tumor responders, the **duration of tumor response** is measured from the date of randomization until the first date of documented disease progression or death due to any cause, whichever occurs first. Duration of tumor response will be censored at the date of the last follow-up visit for tumor responders who are still alive and who have not progressed.

Time to treatment failure is defined as the time from the date of randomization to the date of the first of the following events: early discontinuation of study therapy, progressive disease, or death due to any cause. Time to treatment failure will be censored at the date of the last follow-up visit for patients who did not discontinue early, who are still alive, and who have not progressed.

Time to documented disease progression is defined as the time from the date of randomization to the first date of documented disease progression. Time to documented disease progression will be censored at the date of death for patients who have not had documented disease progression. For patients who are still alive at the time of analysis and who have not had documented disease progression, time to documented disease progression will be censored at the date of the last follow-up visit.

3.9. Safety

Investigators are responsible for monitoring the safety of subjects who have entered this study and for alerting the Principal Investigator of any event that seems unusual, even if this event may be considered an unanticipated benefit to the subject.

The investigator is responsible for appropriate medical care of subjects during the study.

The investigator remains responsible for following, through an appropriate health care option, adverse events that are serious or that caused the subject to discontinue before completing the study. The subject should be followed until the event resolves or is explained. Frequency of follow-up is left to the discretion of the investigator.

3.9.1. Safety Measures

Safety measures that will be used in the study include physical examinations and clinical laboratory tests (haematology and blood chemistries). Patients will be rated for toxicity prior to each cycle using the NCI CTC scale.

3.9.2. Clinical Adverse Events

A clinical trial adverse event is any untoward medical occurrence in a subject administered a pharmaceutical product, without regard to the possibility of a causal relationship. Cases of pregnancy should be reported for tracking purposes. Lack of drug effect is not an adverse event.

Adverse events will be collected after the subject has been enrolled. If a subject experiences an adverse event after the informed consent document is signed (entry) but the subject is never assigned to treatment (enrollment), the event will NOT be reported unless the investigator feels that the event may have been caused by a protocol procedure.

Prior to enrollment, the investigator will note the occurrence and nature of each subject's medical condition(s). During the study, site personnel will again note any change in the condition(s) and the occurrence and nature of any adverse events.

Subjects should be closely followed for adverse events while receiving study therapy and for 30 days after last dose of study therapy in order to detect delayed toxicity. After this period, investigators should only report serious adverse events that are felt to be causally related to study drug therapy or to a protocol procedure.

Events leading to the clinical outcome of death will be included as part of the safety and efficacy analyses for this study, and will not be recorded as adverse events unless the investigator believes the event may have been caused by the study therapy.

3.9.3. Serious Adverse Events (SAE)

3.9.3.1. Definition of SAE

Any of the following would constitute a serious adverse event.

- Death
- Prolonged inpatient hospitalization
- A life-threatening experience (that is, immediate risk of dying)
- Severe or permanent disability
- Congenital anomaly

Neutropenic fever is commonly associated with docetaxel and adriamycin treatment and its incidence has been well characterized, therefore reporting of this event as an SAE is not necessary, unless it results in prolonged hospitalization or death. Neutropenic fever will nonetheless be reported as adverse events.

3.9.3.2. Reporting of SAE

The investigator must record all serious adverse events, regardless of treatment or relationship to study drug, as soon as he/she is informed of the event. Investigators must report immediately to the Principal Investigator any serious adverse events.

3.9.3.3. Follow-up of SAE

Any serious and or unexpected adverse event should be medically well documented and the information made available as soon as possible.

3.10. Discontinuations

The criteria for enrollment must be followed explicitly. If a subject who does not meet enrollment criteria is inadvertently enrolled, that subject should be discontinued from the study and the Principal Investigator contacted.

In addition, patients will be discontinued from the study therapy in the following circumstances:

- Evidence of progressive disease.
- Patient experiences unacceptable toxicity.
- The investigator decides that the patient should be withdrawn. If this decision is because of a serious adverse event or a clinically significant laboratory value, the study drug is to be discontinued and appropriate measures taken.
- The patient requests discontinuation.
- The patient, for any reason, requires another type of tumor treatment. In this case, discontinuation from the study occurs immediately upon introduction of the new treatment.

- The patient becomes pregnant or fails to use adequate birth control (for those patients who are able to conceive).
- The patient is non-compliant with study procedures.
- The investigator, for any reason, stops the study or stops the patient's participation in the study.

Patients who discontinue study therapy early will have post-study follow-up procedures performed as described in the protocol.

All patients who have received at least one dose of study drug will be evaluable for safety analysis. All patients who are registered into the study but fail to receive the first dose of study drug and those who are lost to follow up before disease evaluation will be replaced.

The primary reason for withdrawal will be clearly documented in the subject's medical record and recorded in the CRF. A final evaluation will be completed at the time of discontinuation from the study.

3.11. Clinical laboratory tests and procedures

3.11.1. Pre-study

<u>Prior to study enrollment each patient will have the following assessments:</u>

<u>No more than 4 weeks before study enrollment:</u>

Plain chest X-ray will be performed for baseline tumor assessment. Other imaging (ie CT scans, bone scan or skeletal X-rays) should be performed if clinically indicated, at the discretion of the investigator.

<u>No more than 2 weeks before study enrollment</u>

Medical history and physical examination, including measurements of height, weight, calculation of body surface area, blood pressure, and pulse rate.

- Evaluation of performance status (Karnofsky scale)
- Concomitant medication notation
- Tumor measurement of palpable lesions
- Tumor measurement of skin metastases

<u>Within 7 days before study enrollment:</u>

Haematology

Blood chemistries: bilirubin, ALT, AST, alkaline phosphatase (ALP), creatinine, calcium and electrolytes (sodium, potassium)

A serum pregnancy test for females with childbearing potential.

3.11.2. During the study

3.11.2.1. Efficacy assessment

<u>Prior to each cycle of treatment</u>

Performance status evaluation

Limited medical history and physical examination

Clinical tumor measurement

Radiological assessment to be repeated every 6 weeks where appropriate.

3.11.2.2. Safety assessment

The following tests and procedures will be performed at specific intervals during the study to monitor study drug safety:

- Limited physical examination to include blood pressure, heart rate, weight, height, and calculation of body surface area at each cycle.

- Concomitant medication notation and number of units required for transfusions at every cycle.

- Haematology within 4 days prior to the start of each cycle (except cycle 1 where the 7 days limit before study enrollment applies), and between days 10 to 16 of each cycle. In addition, hematology on day 8 during the first cycle of each chemotherapy drug (ie adriamycin or docetaxel) will be performed as part of pharmacodynamic endpoints.

- Blood chemistries (bilirubin, ALT, AST, creatinine, electrolytes) prior to the start of each cycle.

- Toxicity rating using the NCI CTC scale prior to each cycle.

3.11.3. Laboratory Tests and Results

The local laboratory in National University Hospital will perform all laboratory tests. Investigators must document their review of each laboratory report by signing or initialing and dating each report.

The investigator must evaluate laboratory values that fall outside the clinically accepted reference range or that differ significantly from previous values. Any such significant laboratory changes must be documented and commented on in the CRF.

3.11.4. Post-Study follow-up

3.11.4.1. Efficacy

To obtain meaningful data on time-to-event variables, assessments of disease status will be made at regular intervals after patients discontinue from study therapy. Assessments will continue until documented disease progression, death or 12 months after randomization, whichever occurs first.

During the post-study follow-up period, information will be collected regarding date of disease progression or death. The study will be closed when, in the opinion of the principal investigator, sufficient data have been obtained for completion of the final study manuscript.

3.11.4.2. Safety

After a patient discontinues study therapy, the investigator should make every effort to continue to evaluate the patient for delayed toxicity by clinical and laboratory evaluations as clinically indicated. Every attempt should be made to obtain haematology and chemistry approximately 30 days after the last dose of study treatment. The patient must be followed approximately every 30 days until toxicity resolves.

4. Sample Size and Data Analysis Methods

4.1. Sample Size

We expect 70% of all potentially eligible patients to have non-metastatic locally advanced disease, and 30% to have metastatic disease. Among the patients with non-metastatic disease, we expect approximately 20-25% to have complete clinical response following four cycles of treatment. Therefore, core biopsy to obtain further tumor materials for gene expression studies will no longer be possible. Accordingly, we estimate that we will be able to obtain tumor tissues at all three planned time points in approximately 80-85% of the entire cohort. With this in mind, the target number of patients to be recruited has been set at 100, to ensure that we will have approximately 40 patients in each treatment arm who are fully analyzable in the gene expression studies.

4.2. Data to be analyzed

Clinical Data

Efficacy data that will be analyzed will include:

1. Rates of good clinical response (complete clinical response, minimal residual disease, and partial clinical responses), including confidence intervals.
2. Rates of complete pathological responses.
3. Time to progression

The overall response of the entire patient cohort (Arms A and B combined) will be reported, as we expect the clinical and pathological response rate to the two different treatment schedules to be comparable.

The major analysis to be made include:

1. Comparison of acute gene and protein expression changes in tumor in response to the two different schedules of chemotherapy (ie Arm A versus Arm B).
2. Correlation between clinical response (good clinical response, stable disease and progressive disease) and tumor gene expression and protein expression changes.
3. Correlation between drug pharmacokinetics and treatment toxicity/tumor response.
4. Correlation between patient genotype and drug pharmacokinetics.

No interim analysis is planned.

5. Clinical Pharmacy

Pharmaceutical Information

Docetaxel will be sponsored in part by Aventis. Adriamycin and part of the docetaxel will be purchased from the local manufacturer.

The Cancer Centre pharmacist will be responsible for adequate storage of the study medication according to the manufacturer's recommendations and for dispensing the treatment to the patients.

The study medication must be used in accordance with the protocol and only by the investigator.

The investigator and/or pharmacist must maintain adequate and accurate records, showing the receipt and distribution of all supplies of the study medication.

These records include:

- All accompanying letters that list the batch number of the medication, the quantities received, and the date of reception.
- The drug accountability form that includes the patient's identification, the date of dispensation, each quantity dispensed, and the identity of the dispenser.

6. Special Tests

6.1. Pharmacokinetics studies

Pharmacokinetic sampling will be carried out during the first cycle of administration of each chemotherapy drug (ie adriamycin and docetaxel). A maximum of 8 blood samples for each drug will be taken.

Procedure for collection and processing of blood samples

Blood samples may be collected by venepuncture or via an indwelling peripheral venous line, followed by rapid transfer into heparinised silicon treated glass tubes. The first 1ml blood withdrawn from an indwelling venous line is discarded. Blood sampling that is carried out during chemotherapy infusion must be collected from the contralateral arm of the chemotherapy infusion. If a central venous line is present, blood sampling is allowed from it, provided that this route is not being used for chemotherapy infusion. Care must be taken to collect blood slowly without causing hemolysis.

Blood collection tubes will be pre-labeled for the different time-points. If a sample cannot be collected at the planned time, it will be taken as close as possible to the scheduled time and the exact clock time will be reported in the pharmacokinetics form for computer fitting of the curve.

Assay methods

Docetaxel concentrations will be analyzed using a validated method developed in our HPLC laboratory, using liquid-liquid extraction and reversed phase HPLC. Adriamycin concentrations will be analyzed using fluorescence detection by HPLC methods that have been previously established.

Storage of samples and PK assays

Samples will be stored at -80°C for subsequent bioanalysis. PK assays will be performed in the Department of Pharmacology, National University of Singapore. Individual parameter estimates will be determined by standard compartmental and non-compartmental methods based on WINNONLIN software.

6.2. Genotyping studies

10ml blood will be collected from each subject prior to the start of treatment for genotyping. The blood will be collected into EDTA tubes and stored at 4°C for no longer than 1 week before DNA extraction is carried out. DNA is extracted using the Gentra DNA extraction kit (Gentra Systems, Inc., Minneapolis, USA) and stored at -20°C for subsequent genotyping. Known functional SNPs of *Cyp3A*, *MDR-1 and GSTs* will be characterized. More comprehensive genotyping will be carried out in 'outliers' who have extreme pharmacokinetic parameters, experience exceptional toxicity or tumor response to identify novel functional SNPs using high throughput sequencing techniques.

6.3. Gene expression and protein expression studies and cluster analysis

Core biopsy (Philip Iau) is carried out before chemotherapy, at the end of the first cycle of each chemotherapy drug (adriamycin and docetaxel), and after completion of six cycles of chemotherapy (at lumpectomy or mastectomy, or with core biopsy if surgery is not performed), for a total of four tumor specimens for each patient. In the event that the patient experienced complete remission with no palpable tumour, image-guided biopsy using either mammography or ultra-sonography will be performed as appropriate. Tissues will be snap-frozen for subsequent gene expression studies. Messenger RNA will be extracted from the tissues using standard methods, and subject to expression array analysis in the Genome Institute of Singapore (Edison Liu) using a 15,000-gene oligonucleotide array. For clustering analysis, we will utilise the system developed by Eisen et al that employs standard statistical algorithms to arrange genes according to similarity in pattern of gene expression.[54] Log converted expression data from the cDNAs measured will be subjected to one-dimensional hierachical clustering to compute a dendrogram that assembles all elements into a single tree based on pair-wise calculation of the Pearson correlation coefficient of normalised fluorescence ratios as measures of similarity and average linkage clustering. Results of the clustering will be displayed by TREEVIEW (software available at http://genome-www4.stanford.edu/Microarray/SMD/restech.html). We expect to be able to develop a "training set" using the first 10 patients to establish a pattern of gene expression that can predict response, and validate this using the next 10 patients.

Gene expression in tumors will also be assessed via the analysis of specific epigenetic changes, associated with high-order chromosomal DNA long range interactions, which are fundamental regulatory events which mark any change in gene expression and thus could be used as an indication for abnormal gene expression in a given tissue sample. (Dekker J. Gene regulation in the third dimension. Science. 2008 Mar 28;319(5871):1793-4., Martianov I, Ramadass A, Serra Barros A, Chow N, Akoulitchev A. Repression of the human dihydrofolate reductase gene by a non-coding interfering transcript. Nature. 2007 Feb 8;445(7128):666-70. Epub 2007 Jan 21). Examination of various abnormally expressed genes in annotated samples for renal, colorectal, prostate and breast cancers has confirmed the ability to identify changes in gene expression associated with pathological conditions at the extremely high level of sensitivity and specificity. In this study, paraffinized tumor samples taken before and following treatment will undergo extraction of the chromatin material, which will undergo three consequential enzymatic reactions, at controlled rates of processing the substrate. Polymerase chain reaction (PCR) primers will be utilized to detect the changes in conformation of DNA predicted to be associated with changes in transcription of relevant genes previously described to predict or be associated with treatment response in breast cancer. The final product will be detected on Shimadzu MultiNA.

Proteins from tumor cores will be extracted from the elluent that is obtained during the RNA extraction process. The proteins will be fractionated and profiled using the ProteinChip Array SELDI MS. Changes in protein profiles in response to chemotherapy will be compared between the good and poor responders and correlated with gene expression changes.

6.4 Gene expression studies of peripheral mononuclear cells

2.5 ml of peripheral blood from samples obtained during the pharmacokinetic sampling will be used for this part of the study. The blood will be collected into PAXgene™ tubes (PreAnalytiX), at 2 time points namely, before and 24 hours after exposure to adriamycin and

docetaxel respectively (ie, first and second cycle of chemotherapy). RNA isolation would be done according to the manufacturer's instructions (PAXgene™ Blood RNA kit). The RNA would then be quantified spectrophotometrically and aliqouted for storage at -80^0C. The expected RNA yield is 4-20µg from each sample. A total of 2-5µg of total RNA would then be used to synthesize double-stranded cDNA using T7 dT primers and SuperScript II reverse transcriptase and polymerases (Affymetrix User Manual). cRNA would be synthesized and biotinylated through an in vitro transcription assay and after cleanup (Qiagen) would be quantified spectrophotometrically and a minimum of 15µg used for subsequent experiments. cRNA would then be fragmented for hybridization on the Affymetrix HG-U133A oligonucleotide arrays according to an overnight protocol (Affymetrix User Manual). After washing, arrays would be stained with streptavidin-phycoerythrin (Molecular Porbes) and scanned on a Hewlett Packard scanner. Intensity values would be scaled such that overall intensity for each chip of the same type is equivalent. Intensity for each feature of the array will be captured using the default settings of Affymetrix Microarray Suite software version 5 (MAS 5.0, Affymetrix, Santa Clara, CA) and a single raw expression level for each gene derived from the 20 probe pairs representing each gene will be accomplished by using a trimmed mean algorithm. Raw signals will be log-transformed and probe sets filtered out as deemed absent or no change by MAS 5.0. Standard analytical software will be used to perform the statistical analysis. Unsupervised (hierarchical clustering, principal component analysis) or supervised learning methods (support vector machines, k-nearest neighbor, artificial neural network) would be used to select the most discriminating gene probe sets and ranked according to their discriminating powers.

Epigenetic changes in leukocytes can also reflect the presence of tumor and changes in tumor states (D'Arcy V, Abdullaev ZK, Pore N, Docquier F, Torrano V, Chernukhin I, Smart M, Farrar D, Metodiev M, Fernandez N, Richard C, Delgado MD, Lobanenkov V, Klenova E.The potential of BORIS detected in the leukocytes of breast cancer patients as an early marker of tumorigenesis. Clin Cancer Res. 2006 Oct 15;12(20 Pt 1):5978-86).
Frozen cell pellets from peripheral blood collected before and after treatment with chemotherapy will undergo extraction of the chromatin material, which will undergo three consequential enzymatic reactions, at controlled rates of processing the substrate. Polymerase chain reaction (PCR) primers will be utilized to detect the changes in conformation of DNA predicted to be associated with changes in transcription of relevant genes previously described to predict or be associated with treatment response in breast cancer. The final product will be detected on Shimadzu MultiNA.

7. Study monitoring and data collection

7.1. Data collection

Case Record Forms (CRFs)

All data obtained in the study described in this protocol will be recorded on CRFs. The CRF for each subject will be presented in a folder. The CRF will be completed chronologically and updated regularly in order to reflect the most recent data on the patient included in the study.

Prior to the start of the study, the Investigator will complete a "People authorized to document CRFs" form, showing the signatures and initials of all those who are authorized to make or change entries on the CRFs.

Each CRF must be neatly filled in with a black-inked pen. For each page on which information is entered, the subject number must be recorded. The registration form, the

treatment form and the follow-up status form must be dated and signed by an authorized investigator.

Errors must be corrected by drawing a single line through the incorrect entry and by writing the new value as close as possible to the original. The correction must then be initialed and dated by an authorized person.

Although a research nurse may interview subjects, the investigator must verify that all data entries are accurate and correct, including verification that the subject fulfils the criteria for entrance into the study before study medication is dispensed. Physical examinations have to be performed by a registered medical practitioner.

The End of Treatment Form must be completed for each patient upon completion or withdrawal from the study.

The investigator will add to the subject trial file, after completion of the study, any relevant post-trial information brought to his attention.

7.2. Data Management

Data entry

A data manager will enter data into an electronic database in a password protected, user-designated computer in the office of the CTRG.

Maintenance of patients records

CTRG clinical report forms (CRF) will be used to record data for this study. A copy of the CRF will be kept in the CTRG Office. All records will be kept for a period of 6 years following the date of study closure according to Singapore GCP guidelines.

7.3. Statistical Analysis

To determine correlation between the pharmacokinetics and SNPs of DMEs/drug transporters, two sets of models will be constructed: one to determine the effect of variant alleles on drug PK, and another to build PK-PD relationships. Gene expression and protein expression data will be included in mathematical models with other covariates to generate hypotheses about the contributions of SNPs of DMEs and transporters to the inter-individual pharmacodynamic variability of anticancer drugs.

8. Informed Consent, Ethical Review, and Regulatory Considerations

8.1. Informed Consent

The informed consent document will be used to explain the risks and benefits of study participation to the patient in layman terms before the patient is entered into the study.

The investigator is responsible to see that informed consent is obtained from each patient or legal representative and to obtain the appropriate signatures and dates on the informed consent document prior to the performance of any protocol procedures and prior to the administration of study drug.

As used in this protocol, the term "informed consent" includes all consent and/or assent given by patients and their legal representatives.

8.2. Patient information

The responsible physician will inform the patient about the background and current knowledge of the treatment under study with special reference to known activity and toxicity. The patient will be told about the investigative nature of this treatment and in particular, the randomization process involved in this study. The patient will be told of his or her right to withdraw from the study at any time without any penalty with regards to the continuation of care at this institution and by the same physicians as he chooses. The patient will be told that tissue and blood samples obtained for genetic studies will be assigned unique patient numbers (UPN) to ensure patient confidentiality.

8.3. Ethical Review

Approval of the protocol and the informed consent document will be obtained from the institution's ethical review board before the study may begin.

The investigator will supply the following to the study site's ethical review board(s):

- The study protocol
- The current Clinical Investigator's Brochure or package labeling and updates during the course of the study
- Informed consent document
- Relevant curricula vitae

8.4. Regulatory Considerations

This study will be conducted in accordance with the ethical principles stated in the most recent version of the Singapore guidelines on good clinical practice (GCP).

9. References

1. Lander ES, Linton LM, Birren B, Nusbaum C, Zody MC, Baldwin J et al. Initial sequencing and analysis of the human genome. Nature 2001; 409: 860-921.
2. Huang JX, Mehrens D, Wiese R, Lee S, Tam SW, Daniel S et al. High-throughput genomic and proteomic analysis using microarray technology. Clin Chem 2001; 47: 1912-6.
3. Liotta LA, Liu ET. Essentials of molecular biology: genomics of cancer. Cancer: Principles & Practice of Oncology, 6th Edition; VT Devita, S Hellman, SA Rosenberg 2001; 1: 17-41.
4. Tan AR, Swain SM. Adjuvant chemotherapy for breast cancer: an update. Semin Oncol 2001; 28: 359-76.
5. Tallman MS, Andersen JW, Schiffer CA, Appelbaum FR, Feusner JH, Ogden A et al. All-trans-retinoic acid in acute promyelocytic leukemia. N Engl J Med 1997; 337: 1021-8.
6. Harvey JM, Clark GM, Osborne CK, Allred DC. Estrogen receptor status by immunohistochemistry is superior to the ligand-binding assay for predicting response to adjuvant endocrine therapy in breast cancer. J Clin Oncol 1999; 17: 1474-81.
7. Muss HB, Thor AD, Berry DA, Kute T, Liu ET, Koerner F et al. c-erbB-2 expression and response to adjuvant therapy in women with node- positive early breast cancer. N Engl J Med 1994; 330: 1260-6.
8. Monzo M, Rosell R, Sanchez JJ, Lee JS, O'Brate A, Gonzalez-Larriba JL et al. Paclitaxel resistance in non-small-cell lung cancer associated with beta-tubulin gene mutations. J Clin Oncol 1999; 17: 1786-93.
9. Esteller M, Garcia-Foncillas J, Andion E, Goodman SN, Hidalgo OF, Vanaclocha V et al. Inactivation of the DNA-repair gene MGMT and the clinical response of gliomas to alkylating agents. N Engl J Med 2000; 343: 1350-4.
10. Goldman JM, Melo JV. Targeting the BCR-ABL tyrosine kinase in chronic myeloid leukemia. N Engl J Med 2001; 344: 1084-6.
11. Druker BJ, Talpaz M, Resta DJ, Peng B, Buchdunger E, Ford JM et al. Efficacy and safety of a specific inhibitor of the BCR-ABL tyrosine kinase in chronic myeloid leukemia. N Engl J Med 2001; 344: 1031-7.
12. Shoemaker DD, Schadt EE, Armour CD, He YD, Garrett-Engele P, McDonagh PD et al. Experimental annotation of the human genome using microarray technology. Nature 2001; 409: 922-7.
13. Wu TD. Analysing gene expression data from DNA microarrays to identify candidate genes. J Pathol 2001; 195: 53-65.
14. Wang E, Miller LD, Ohnmacht GA, Liu ET, Marincola FM. High-fidelity mRNA amplification for gene profiling. Nat Biotechnol 2000; 18: 457-9.
15. Powles TJ, Dowsett M, Sotiriou C, Simon R, Zhao Y, Liu E. Use of cDNA microarrays to estimate gene expression profiles from FNA samples of breast cancer. Proc ASCO 2001; 20: 35a.
16. Liotta LA, Kohn EC, Petricoin EF. Clinical proteomics: personalized molecular medicine. Jama 2001; 286: 2211-4.
17. Petricoin EF, Zoon KC, Kohn EC, Barrett JC, Liotta LA. Clinical proteomics: translating benchside promise into bedside reality. Nat Rev Drug Discov 2002; 1: 683-95.
18. Ratain MJ. Body-surface area as a basis for dosing of anticancer agents: science, myth, or habit? J Clin Oncol 1998; 16: 2297-8.
19. Gurney H. Dose calculation of anticancer drugs: a review of the current practice and introduction of an alternative. J Clin Oncol 1996; 14: 2590-611.
20. Nielsen D, Dombernowsky P, Larsen SK, Hansen OP, Skovsgaard T. Epirubicin or epirubicin and cisplatin as first-line therapy in advanced breast cancer. A phase III study. Cancer Chemother Pharmacol 2000; 46: 459-66.
21. de Jongh FE, Verweij J, Loos WJ, de Wit R, de Jonge MJ, Planting AS et al. Body-surface area-based dosing does not increase accuracy of predicting cisplatin exposure. J Clin Oncol 2001; 19: 3733-9.
22. Roses AD. Pharmacogenetics and the practice of medicine. Nature 2000; 405: 857-65.
23. Takahashi H, Echizen H. Pharmacogenetics of warfarin elimination and its clinical implications. Clin Pharmacokinet 2001; 40: 587-603.
24. Ulrich CM, Yasui Y, Storb R, Schubert MM, Wagner JL, Bigler J et al. Pharmacogenetics of methotrexate: toxicity among marrow transplantation patients varies with the methylenetetrahydrofolate reductase C677T polymorphism. Blood 2001; 98: 231-4.
25. Relling MV, Hancock ML, Rivera GK, Sandlund JT, Ribeiro RC, Krynetski EY et al. Mercaptopurine therapy intolerance and heterozygosity at the thiopurine S-methyltransferase gene locus. J Natl Cancer Inst 1999; 91: 2001-8.
26. Hoffmeyer S, Burk O, von Richter O, Arnold HP, Brockmoller J, Johne A et al. Functional polymorphisms of the human multidrug-resistance gene: multiple sequence variations and correlation of one allele with P-glycoprotein expression and activity in vivo. Proc Natl Acad Sci U S A 2000; 97: 3473-8.

27. van Kuilenburg AB, Muller EW, Haasjes J, Meinsma R, Zoetekouw L, Waterham HR et al. Lethal outcome of a patient with a complete dihydropyrimidine dehydrogenase (DPD) deficiency after administration of 5-fluorouracil: frequency of the common IVS14+1G>A mutation causing DPD deficiency. Clin Cancer Res 2001; 7: 1149-53.
28. Weinstein JN, Myers TG, O'Connor PM, Friend SH, Fornace AJJ, Kohn KW et al. An information-intensive approach to the molecular pharmacology of cancer. Science 1997; 275: 343-9.
29. Hughes TR, Marton MJ, Jones AR, Roberts CJ, Stoughton R, Armour CD et al. Functional discovery via a compendium of expression profiles. Cell 2000; 102: 109-26.
30. Stegmaier K, Ross KN, Colavito SA, O'Malley S, Stockwell BR, Golub TR. Gene expression-based high-throughput screening(GE-HTS) and application to leukemia differentiation. Nat Genet. 2004; 36: 257-63.
31. Cheok MH, Yang W, Pui CH, Downing JR, Cheng C, Naeve CW et al. Treatment-specific changes in gene expression discriminate in vivo drug response in human leukemia cells. Nat Genet. 2003; 34: 85-90.
32. Holleman A, den Boer ML, Cheok MH, Janka-Schaub GE, Veerman AJP, Pieters R et al. Gene expression profiles associated with drug resistance in paediatric acute lymphoblastic leukaemia blasts. Proc Am Assoc Cancer Res 2003; 44: 310.
33. Whitney AR, Diehn M, Popper SJ, Alizadeh AA, Boldrick JC, Relman DA et al. Individuality and variation in gene expression patterns in human blood. Proc Natl Acad Sci U S A. 2003; 100: 1896-901.
34. DePrimo SE, Wong LM, Khatry DB, Nicholas SL, Manning WC, Smolich BD et al. Expression profiling of blood samples from an SU5416 Phase III metastatic colorectal cancer clinical trial: a novel strategy for biomarker identification. BMC Cancer 2003; 3: 3.
35. Gaedigk A. Interethnic differences of drug-metabolizing enzymes. Int J Clin Pharmacol Ther 2000; 38: 61-8.
36. Lin HJ, Han CY, Lin BK, Hardy S. Ethnic distribution of slow acetylator mutations in the polymorphic N-acetyltransferase (NAT2) gene. Pharmacogenetics 1994; 4: 125-34.
37. Aynacioglu AS, Sachse C, Bozkurt A, Kortunay S, Nacak M, Schroder T et al. Low frequency of defective alleles of cytochrome P450 enzymes 2C19 and 2D6 in the Turkish population. Clin Pharmacol Ther 1999; 66: 185-92.
38. Shu Y, Wang LS, Xu ZH, He N, Xiao WM, Wang W et al. 5-hydroxylation of omeprazole by human liver microsomal fractions from Chinese populations related to CYP2C19 gene dose and individual ethnicity. J Pharmacol Exp Ther 2000; 295: 844-51.
39. Xiao ZS, Goldstein JA, Xie HG, Blaisdell J, Wang W, Jiang CH et al. Differences in the incidence of the CYP2C19 polymorphism affecting the S-mephenytoin phenotype in Chinese Han and Bai populations and identification of a new rare CYP2C19 mutant allele. J Pharmacol Exp Ther 1997; 281: 604-9.
40. Attitallah S, Berard M, Belkahia C, Bechtel YC, Bechtel PR. Similarities and/or dissimilarities of CYP2D6 polymorphism in three Tunisian ethnic groups: Arabs, Berbers, Numides. Therapie 2000; 55: 355-60.
41. Teh LK, Ismail R, Yusoff R, Hussein A, Isa MN, Rahman AR. Heterogeneity of the CYP2D6 gene among Malays in Malaysia. J Clin Pharm Ther 2001; 26: 205-11.
42. Beutler E, Gelbart T, Demina A. Racial variability in the UDP-glucuronosyltransferase 1 (UGT1A1) promoter: a balanced polymorphism for regulation of bilirubin metabolism? Proc Natl Acad Sci U S A 1998; 95: 8170-4.
43. Lampe JW, Bigler J, Horner NK, Potter JD. UDP-glucuronosyltransferase (UGT1A1*28 and UGT1A6*2) polymorphisms in Caucasians and Asians: relationships to serum bilirubin concentrations. Pharmacogenetics 1999; 9: 341-9.
44. Ameyaw MM, Regateiro F, Li T, Liu X, Tariq M, Mobarek A et al. MDR1 pharmacogenetics: frequency of the C3435T mutation in exon 26 is significantly influenced by ethnicity. Pharmacogenetics 2001; 11: 217-21.
45. Sata F, Sapone A, Elizondo G, Stocker P, Miller VP, Zheng W et al. CYP3A4 allelic variants with amino acid substitutions in exons 7 and 12: evidence for an allelic variant with altered catalytic activity. Clin Pharmacol Ther 2000; 67: 48-56.
46. Sullivan-Klose TH, Ghanayem BI, Bell DA, Zhang ZY, Kaminsky LS, Shenfield GM et al. The role of the CYP2C9-Leu359 allelic variant in the tolbutamide polymorphism. Pharmacogenetics 1996; 6: 341-9.
47. Goldstein JA, Ishizaki T, Chiba K, de Morais SM, Bell D, Krahn PM et al. Frequencies of the defective CYP2C19 alleles responsible for the mephenytoin poor metabolizer phenotype in various Oriental, Caucasian, Saudi Arabian and American black populations. Pharmacogenetics 1997; 7: 59-64.
48. Mathew J, Basheeruddin K, Prabhakar S. Differences in frequency of the deletion polymorphism of the angiotensin-converting enzyme gene in different ethnic groups. Angiology 2001; 52: 375-9.
49. Cao K, Hollenbach J, Shi X, Shi W, Chopek M, Fernandez-Vina MA. Analysis of the frequencies of HLA-A, B, and C alleles and haplotypes in the five major ethnic groups of the United States reveals high levels of diversity in these loci and contrasting distribution patterns in these populations. Hum Immunol 2001; 62: 1009-30.

50. Yap WS, Chan CC, Chan SP, Wang YT. Ethnic differences in anthropometry among adult Singaporean Chinese, Malays and Indians, and their effects on lung volumes. Respir Med 2001; 95: 297-304.
51. Fisher B, Bryant J, Wolmark N, Mamounas E, Brown A, Fisher ER et al. Effect of preoperative chemotherapy on the outcome of women with operable breast cancer. J Clin Oncol 1998; 16: 2672-85.
52. Powles TJ, Hickish TF, Makris A, Ashley SE, O'Brien ME, Tidy VA et al. Randomized trial of chemoendocrine therapy started before or after surgery for treatment of primary breast cancer. J Clin Oncol 1995; 13: 547-52.
53. Chang J, Powles TJ, Allred DC, Ashley SE, Clark GM, Makris A et al. Biologic markers as predictors of clinical outcome from systemic therapy for primary operable breast cancer. J Clin Oncol 1999; 17: 3058-63.
54. Eisen MB, Spellman PT, Brown PO, Botstein D. Cluster analysis and display of genome-wide expression patterns. Proc Natl Acad Sci U S A 1998; 95: 14863-8.

10 Appendix
10.1 AJCC Staging Criteria

Breast cancer staging information

The American Joint Committee on Cancer (AJCC) staging system provides a strategy for grouping patients with respect to prognosis. Therapeutic decisions are formulated in part according to staging categories but primarily according to tumor size, lymph node status, estrogen- and progesterone-receptor levels in the tumor tissue, menopausal status, and the general health of the patient.

The AJCC has designated staging by TNM classification.

TNM definitions

Primary tumor (T):

TX: Primary tumor cannot be assessed
T0: No evidence of primary tumor
Tis: Carcinoma in situ; intraductal carcinoma, lobular carcinoma in situ, or Paget's disease of the nipple with no associated tumor
Note: Paget's disease associated with a tumor is classified according to the size of the tumor
T1: Tumor 2.0 cm or less in greatest dimension
- T1mic: Microinvasion 0.1 cm or less in greatest dimension
- T1a: Tumor more than 0.1 but not more than 0.5 cm in greatest dimension
- T1b: Tumor more than 0.5 cm but not more than 1.0 cm in greatest dimension
- T1c: Tumor more than 1.0 cm but not more than 2.0 cm in greatest dimension

T2: Tumor more than 2.0 cm but not more than 5.0 cm in greatest dimension
T3: Tumor more than 5.0 cm in greatest dimension
T4: Tumor of any size with direct extension to (a) chest wall or (b) skin, only as described below
Note: Chest wall includes ribs, intercostal muscles, and serratus anterior muscle but not pectoral muscle
- T4a: Extension to chest wall
- T4b: Edema (including peau d'orange) or ulceration of the skin of the breast or satellite skin nodules confined to the same breast
- T4c: Both of the above (T4a and T4b)
- T4d: Inflammatory carcinoma*

* Note: Inflammatory carcinoma is a clinicopathologic entity characterized by diffuse brawny induration of the skin of the breast with an erysipeloid edge, usually without an underlying palpable mass. Radiologically there may be a detectable mass and characteristic thickening of the skin over the breast. This clinical presentation is due to tumor embolization of dermal lymphatics with engorgement of superficial capillaries.

Regional lymph nodes (N):

NX: Regional lymph nodes cannot be assessed (e.g., previously removed)
N0: No regional lymph node metastasis
N1: Metastasis to movable ipsilateral axillary lymph node(s)
N2: Metastasis to ipsilateral axillary lymph node(s) fixed to each other or to other structures
N3: Metastasis to ipsilateral internal mammary lymph node(s)

Pathologic classification (pN):

pNX: Regional lymph nodes cannot be assessed (not removed for pathologic study or previously removed)
pN0: No regional lymph node metastasis
pN1: Metastasis to movable ipsilateral axillary lymph node(s)
- pN1a: Only micrometastasis (none larger than 0.2 cm)
- pN1b: Metastasis to lymph node(s), any larger than 0.2 cm
- pN1bi: Metastasis in 1 to 3 lymph nodes, any more than 0.2 cm and all less than 2.0 cm in greatest dimension

pN1bii: Metastasis to 4 or more lymph nodes, any more than 0.2 cm and all less than 2.0cm in greatest dimension
pN1biii: Extension of tumor beyond the capsule of a lymph node metastasis less than 2.0 cm in greatest dimension
pN1biv: Metastasis to a lymph node 2.0 cm or more in greatest dimension
pN2: Metastasis to ipsilateral axillary lymph node(s) fixed to each other or to other structures
pN3: Metastasis to ipsilateral internal mammary lymph node(s)

Distant metastasis (M):

MX: Presence of distant metastasis cannot be assessed
M0: No distant metastasis
M1: Distant metastasis present (includes metastasis to ipsilateral supraclavicular lymph nodes)

AJCC stage groupings
Stage 0
Tis, N0, M0

Stage I
T1,* N0, M0

*T1 includes T1mic

Stage IIA
T0, N1, M0
T1,* N1,** M0
T2, N0, M0

*T1 includes T1mic
**The prognosis of patients with pN1a disease is similar to that of patients with pN0 disease.

Stage IIB
T2, N1, M0
T3, N0, M0

Stage IIIA
T0, N2, M0T1,* N2, M0
T2, N2, M0
T3, N1, M0
T3, N2, M0

*T1 includes T1mic

Stage IIIB
T4, Any N, M0
Any T, N3, M0

Stage IV
Any T, Any N, M1

10.2. Karnofsky Performance Scale

Able to carry on normal activity; no special care needed	100	Normal, no complaints; no evidence of disease
	90	Able to carry on normal activity; minor signs or symptoms of disease
	80	Normal activity with effort; some signs or symptoms of disease
Unable to work; able to live at home and care for most personal needs; a varying amount of assistance is needed	70	Cares for self; unable to carry on normal activity or to do active work
	60	Requires occasional assistance but is able to care for most of his needs
	50	Requires considerable assistance and frequent care
Unable to care for self; requires equivalent of institutional or hospital care; disease may be progressing rapidly	40	Disabled; requires special care and assistance
	30	Severely disabled; hospitalization is indicated although death not imminent
	20	Very sick; hospitalization necessary; active supportive treatment is necessary
	10	Moribund; fatal processes progressing rapidly
	0	Dead

10.3. Patient Information Sheet and Consent From

<u>INFORMATION TO THE PATIENT (original version – superceded by latest IRB-approved version)</u>

Gene expression profiles of breast cancer treated with sequential adriamycin and docetaxel in relation to tumor response.

Introduction
This document gives a description of the study in which you are being asked to participate.

Purpose and Design of Study
We would like you to take part in a research study using two drugs, called adriamycin and docetaxel, in the treatment of breast cancer. Both adriamycin and docetaxel have been used successfully, either alone, or in combination in the treatment of breast cancer, with good results. A total of twenty-six patients in the National University Hospital, Singapore will take part in the study over a period of about 18 months.

By participating in this study, you will receive adriamycin alternating with docetaxel for a total of six cycles, starting with either adriamycin or docetaxel. The purpose of our study is to determine whether genetic changes in the cancer may be used to predict how your cancer will respond to chemotherapy, and whether there is a difference to cancer response and gene changes in your cancer when adriamycin or docetaxel is given before the other. We are also studying how your body reacts to each of these two chemotherapy drugs and how these reactions relate to your genes.

Description of study
Chemotherapy is given once every 3 weeks, defined as a cycle. You will be receiving either adriamycin or docetaxel during each cycle. If you agree to enter the study, you will be 'randomised' to either starting with adriamycin or with docetaxel. 'Randomisation' means that neither you nor your doctor will be able to choose which drug you would start with. You will have the same chance of starting with adriamycin, or with docetaxel. Adriamycin will be administered as a 15-minute infusion, while docetaxel will be administered as a 1-hour infusion. The treatment will be given in the outpatient clinic. You will receive at least **two** cycles of treatment unless your tumor progresses during treatment or you experience serious side effects.

Your doctor will examine you before you enter this study and before each treatment. You will have the routine physical examinations, blood tests (before the treatment, before each cycle, once to twice during each cycle, and when clinically indicated); chest X-ray (baseline); CT-scans (when clinically indicated). These tests are to ensure your safety and for the doctors to monitor your progress during the treatment.

About 8 samples of blood will be collected, each consisting of 1 teaspoon, when you receive the first cycle of each chemotherapy drug (ie, adriamycin or docetaxel). This is done through a plastic tube inserted into one of your arm veins so that discomfort of needle pricks are minimised. These studies would help us learn how your body handles the chemotherapy drug. An additional tablespoonful of blood will be collected before you start the first cycle of treatment for gene studies. These are studies to understand genetic factors that may affect how your body reacts to the chemotherapy drugs.

A sample of your breast cancer will be obtained from you before treatment, after you receive the first cycle of each chemotherapy drug, and after you complete the treatment, through a routine procedure in the clinic called a core biopsy. This is a procedure whereby a small needle is used to take a sample of cancer tissue from you. This procedure is safe, and the main side effect is that of slight pain and bleeding. The purpose of taking these samples is to allow doctors to determine your cancer response to treatment, as well as to obtain genetic materials to study changes.

Potential adverse effects
Both adriamycin and docetaxel have been used extensively in the treatment of advanced breast cancer.
Side effects will probably include:
- temporary lowering of the white blood cells, sometimes accompanied by fever and shivering. If these occur, you should immediately tell your doctor so that the necessary treatment (antibiotics) can be started and so that the next administration of the medicine is postponed,
- total hair loss,
- allergic reaction,
- nausea and vomiting; these side effects may be alleviated using anti-vomiting medications,
- constipation, diarrhoea,
- tingling in the limbs,
- inflammation of the mucous membranes of the mouth,
- pain at the place where the injection needle was inserted,
- impaired heart function with large cumulative doses of adriamycin; however, the total doses of adriamycin you will receive by participating in this study is low and is very unlikely to result in this complication.

We advise you to check your body temperature regularly by taking your temperature by the mouth or under one arm. If you notice any of these symptoms or any other clinical signs such as a severe fatigue or fever, **please phone your doctor**. He/she will tell you what must be done and may, if the case arises, modify or change the treatment. Similarly, it will be important to inform your doctor of any medicine that you take during this treatment, even if it seems insignificant to you. Women of childbearing potential must agree to have adequate contraception for the duration of the study and for 60 days after last treatment.

Possible benefits
Both adriamycin and docetaxel have proven high activity against your cancer, and there is a high chance of shrinking your tumour. If your tumour was too large for surgery, this treatment has a good chance of reducing the size to the point of being able to go for surgery. However, although we know that chemotherapy can improve the evolution of your disease, there is individual variation to response to this treatment, and we cannot guarantee with certainty that you will have any benefit. In fact, we are hoping to be able to use the information derived from this study to predict who will benefit from this treatment most, and what doses of drugs to give for optimum effectiveness and minimal side effects.

Alternatives

If you choose not to participate in this study, you may continue to receive standard regimens of chemotherapy for shrinking the tumour before surgery. Your decision not to participate in this study will in no way affect your continued care in this institution with your physician.

Costs

You will be responsible for paying 50% of the costs of docetaxel, full costs of adriamycin, routine blood tests, x-rays, scans, other laboratory tests and medical care.

You will not be paid for your participation in this study.

Confidentiality

All data obtained during the study concerning you will be treated as confidential and only revealed to the legal or health authorities if they so require. No information bearing your name will be supplied to any person whatsoever, apart from the doctors participating in the study. You will not be individually identified in any report and/or publication based on this study. They may be checked in accordance with the regulations currently in effect. Your genetic material will be kept safe in our academic institution and will not be released to industries for profit making without seeking clearance from our ethical committees. On the other hand, if there is intellectual property arising from this study, you will not have any claim on this.

Patient's protection

Protection of patient:

This study is organised in accordance with the International Consensus of Harmonization – Good Clinical Practice Guidelines (ICH-GCP). If you follow the instructions of the doctor in charge of this study and you are injured as a result of your participation in this study, the National University Hospital will pay for the treatment of that injury. Payment for management of the expected consequences of your treatment, including the management of severe nausea/vomiting or hospitalisation due to fever, will not be provided by the National University Hospital.

Ethics Committee

This protocol was submitted for examination by the National University Hospital Research and Ethics Committee (NUH REC) whose task is to check that the conditions required for your protection and the respect of your rights have been complied with. The Committee gave its approval before the beginning of the study.

Your rights

You are free to decide whether or not to participate in this study. You may refuse and, even if you accept, you may withdraw from the study at any time without having to give the reason for your decision. Your refusal or subsequent withdrawal will have no effect on the future management of your disease with your doctor. If you so wish, your doctor will continue to treat you with the best means available. Your relationship with the medical team will be completely unaffected by your decision.

Your doctor may also stop the study at any time without your consent if they feel there is a reason to do this. If any important information becomes available during this study, you and your hospital doctor will be informed of it.

Whatever your decision, we thank you for the attention you have paid to this information sheet.

If you have any questions regarding this study, please ask your doctor.

For an independent opinion regarding the trial and your rights, please contact a member of the NUH REC (Attn: Ms Ms Emily Cheong) at telephone 772 5927.

Patient Informed Consent

Gene expression profiles of breast cancer treated with sequential adriamycin and docetaxel in relation to tumor response.

- This trial has been explained to me in a language ______________ (state the language used) I understand by ______________ (name of translator) on ______________ (date).

- I have received, read and understood the **Patient Information Sheet** for the above study. I have also received an adequate explanation of this clinical study, its purposes, risks and my rights as a patient and what is done to me. I have been given every opportunity to ask questions before making my decision. I may request additional information at any time from the investigator.

- I know that the decision to take part in this study is voluntary and that I have the right to change my mind at any time during the course of the study without penalty or loss of benefits to which I am otherwise entitled. I shall then inform the investigator.

- I also understand that access to relevant information from my patient notes may be required as part of the study, and that data collected during the study could be checked by Health Authorities and sponsor's representatives according to current legislation.
  I am aware that I will not be identifiable from such data extracted from my patient notes and all data will be processed with the strictest confidence.

- My consent does not relieve the investigator from his legal obligations.

- I agree to inform my doctor of any medicine that I take during this study.

- I have been given a copy of this document and I was told that one copy will be held in confidence by the investigator of this study.

On this basis, I consent to take part in this study.

______________	______________	______________
Name of subject	Name of Investigator/ Research staff	Name of witness
______________	______________	______________
Signature of subject	Signature of Investigator/ Research staff	Signature of witness
______________	______________	______________
Date	Date	Date

10.4. Evaluation and visit schedule

	Pre-treatment	Cycles 1 and 2			Cycles 3 to 6		Withdrawal or End of Treatment	Post Study (30 Days post last dose)
		Day 1	Day 8	Day 15	Day 1	Days 10 -16		
Inclusion/Exclusion criteria	X							
Informed consent	X							
Medical history [a]	X							
Physical examination BSA, KPS, V/Signs [a]	X							X
Limited medical history, physical examination, BSA, KPS, V/signs [b]		X			X			
Concomitant medications [a]	X	X	X	X	X	X	X	
Adverse events evaluation [a] (NCI CTC grading)	X	X	X	X	X	X	X	X [h]
Clinical tumor measurement [c] (Palpable lesions and skin metastasis)	X	X			X			
Chest x-ray [d]	X							
CT scans, bone scan & skeletal x-ray [d]	X					X [e]		
Haematology (FBC) [f]	X	X	X	X	X	X		X
Chemistries (Sp#1, CAP#1) [f]	X	X			X			X
B-HCG (patient with child bearing potential) [f]	X							
Tumor core biopsy [g]	X			X			X	
Genotyping adriamycin & docetaxel sampling		X						

[a] not more than 2 weeks before study enrollment.
[b] to be performed prior to each cycle, from cycle 2 onwards.
[c] within 2 weeks of study enrollment. Subsequent assessments to be done every 3 weeks.
[d] within 4 weeks prior to study enrollment. Chest x-ray is mandatory, all other imaging studies (CT scans, bone scans, skeletal x-rays should be performed if clinically indicated, at the discretion of the investigator. The same clinical evaluation and radiological studies must be consistently used for subsequent tumour assessment.
[e] radiological assessment to be repeated every 6 weeks, where appropriate.
[f] within 7 days prior to study enrollment. From cycle 2, haematology to be done within 4 days prior to the start of cycles.
[g] to be performed before treatment, after cycles 1 and 2 (post nadir, prior to the next cycle), and at withdrawal or completion of the Treatment, for a total of four biopsies. The final biopsy may be obtained at surgery.
[h] all study drugs related toxicities must be followed appropriately every 30 days till resolved.
