# Supplementary material for: High-Throughput Mutation Profiling Changes before and 3 Weeks after Chemotherapy in Newly Diagnosed Breast Cancer Patients
Source: PLoS One. 2015 Dec 2;10(12):e0142466. doi: 10.1371/journal.pone.0142466 (PMC4667877; doi:10.1371/journal.pone.0142466)
Supplement: S1 Table — (PDF) [file pone.0142466.s003.pdf]

S1 Table. OncoCarta v1.0 : 238 mutations from 19 oncogenes

Oncogene (number of mutations)

ABL1 (14)	*EGFR* (55)	*HRAS* (10)	*NRAS* (19)
AKT1 (7)	*ERBB2* (7)	*JAK2* (1)	*PDGFRA* (11)
AKT 2 (2)	*FGFR1* (2)	*KIT* (32)	*PIK3CA* (14)
BRAF (25)	*FGFR3* (7)	*KRAS* (16)	*RET* (6)
CDK-4 (2)	*FLT3* (3)	*MET* (5)	
